# Investigation into Polyphenol Profile and Biological Activities of Enriched Persimmon/Apple Smoothies during Storage

**DOI:** 10.3390/foods12173248

**Published:** 2023-08-29

**Authors:** Katarzyna Angelika Gil, Paulina Nowicka, Aneta Wojdyło, Carlo Ignazio Giovanni Tuberoso

**Affiliations:** 1 Department of Life and Environmental Sciences, University of Cagliari, Cittadella Universitaria di Monserrato, S.P. Monserrato-Sestu km 0.700, 09042 Monserrato, Italy; kasiagil8a@gmail.com; 2 Department of Fruit, Vegetable and Plant Nutraceutical Technology, Wrocław University of Environmental and Life Sciences, 37 Chełmońskiego Street, 51-630 Wroclaw, Poland; paulina.nowicka@upwr.edu.pl

**Keywords:** *Diospyros kaki* L., smoothies, storage, polyphenols, antioxidant activity, digestive enzymes inhibition, LC-PDA/MS QTof, sensory evaluation

## Abstract

Smoothies are becoming an increasingly popular product as a healthy alternative to snacks. The consumer expects from this product that, apart from its nutritional value, it will also be qualitatively stable during storage. Therefore, in this study, original smoothies obtained with persimmon fruit puree and apple juice (Dk/Md) enriched with *Arbutus unedo* fruits, *Myrtus communis* purple berry extract, *Acca sellowiana*, and *Crocus sativus* petal juice were evaluated for their polyphenol composition, antioxidant activity, and inhibition on targeted digestive enzymes, over six months of storage. The amount of polyphenols evaluated by UPLC-PDA analysis decreased in six months from 23.5% for both Dk/Md and enriched *C. sativus* smoothies to 42.5% for enriched *A. sellowiana*, with anthocyanins the most sensitive compounds (71.7–100% loss). Values of antioxidant assays generally strongly decreased during the first three months (up to ca. 60%) and to a lesser extent in the following three months (0.4–27%). In addition, inhibitory activity on α-amylase, α-glucosidase, and pancreatic lipase, especially on the last two enzymes, was negatively affected by time storage. The outcome of this study indicates that persimmon fruit is a good option for producing smoothies, and enrichment with other plant extracts can enhance the bioactive compound content and biological activities. It is recommended that appropriate storage strategies to preserve the properties of those smoothies should be developed.

## 1. Introduction

Persimmon (*Diospyros kaki* L.) fruit is a tropical, sweet, fleshy-fibrous fruit that, when ripe, contains thick jelly pulp encased in a waxy, thin-skinned shell [1]. Commercially, persimmon is classified into four types depending on the effect of pollination on the flesh colour, the presence of seeds, and their pattern of astringency loss: pollination-constant non-astringent, pollination-variant non-astringent, pollination-variant astringent, and pollination-constant astringent [2,3]. Persimmon fruits are a source of many bioactive compounds, like polyphenols, vitamins (vitamins B1, B2, B3, A, E, K, and C), minerals (calcium and potassium) [4], sugars (sucrose, glucose, and fructose), carotenoids, tocopherols [5], and dietary fibres [6]. Generally, the main polyphenols in these fruits are flavonols (quercetin and rutin), hydroxycinnamic acids (caffeic, *p*-coumaric, and ferulic acids), hydroxybenzoic acids (gallic, vanillic, and syringic acids) and proanthocyanidin (catechin) [7]. Due to the content of those compounds, persimmon fruits show interesting nutritional properties and antioxidant, anticancer, anti-ageing, and cytotoxic activities [8], provide a natural defence against free radicals [9,10], and have a cholesterol-lowering effect [11].

The persimmon fruit is mainly eaten fresh but can be frozen, canned, or dried and is sometimes used in Oriental cooking. Usually, whole fruits and slices are dried to obtain dried persimmon products. Moreover, ripe, non-astringent, and sweet fruits can be used as a sweetening ingredient in fruity ice creams and baked products, as well as material for the preparation of other products such as nectars, jams, and jellies [1]. Persimmon pulp devoid of peel can be used to make purée, juice, and sherbets [12]. In addition, by-products (e.g., skin, peduncle, and seeds) generated by new products based on persimmon are rich in bioactive compounds and can be used in the food and pharmaceutical industries [12]. Peel polyphenol content contributes to the prevention of oxidative stress-related diseases such as diabetes [9].

The food industry continues to pursue innovative food products with extended shelf life while avoiding the use of preservatives. Maintaining the properties of fresh fruit-based juices and smoothies is a crucial aspect of their use. The traditional and primary technology for processing these products is thermal processing with heat treatments between 60 and 100 °C. Thermal processing is currently the most cost-effective means of ensuring microbial safety and enzyme deactivation [13] and can have a positive effect on enhancing the number of phytochemicals (e.g., phenolic compounds) that can be released from the food matrix in the gastrointestinal tract, improving both their bioaccessibility and bioavailability [14]. Alternative treatments, such as high-pressure processing [15,16] and ultrasound treatments, are increasingly applied [17]. The impact of these treatments on storage stability and effects on physicochemical parameters, phytochemicals, and biological activities were evaluated on different smoothies such as melon-based smoothies enriched with herb extracts [18], blueberry and cranberry smoothies [19], mango smoothies [20], and pomegranate smoothies [21].

Additionally, health-conscious consumers prioritize the equilibrium between the nutritional value and taste of food items. Currently, a range of plant-based snacks has been prepared using different methods offering diverse health benefits. Nevertheless, snacking preferences constantly evolve in response to changing consumer needs.

Recently, it was investigated the effect of apple juice enrichment with selected plant materials [22] and the potential health-promoting activities of innovative smoothies obtained with strawberry tree fruit puree and apple juice enriched with different plant materials [23]. Up until now, the blending of persimmon (Dk) with apple (*Malus domestica*, Md) juice for preparing smoothies and enriching them with different plant materials (*Arbutus unedo* Au (Au) fruits, *Myrtus communis* (Mc) purple berries extract, *Acca sellowiana* (As), and *Crocus sativus* (Cs) petal juice) has not been explored. Consequently, the bioactive profiles of smoothies based on persimmon have not yet been investigated. These aspects of the study hold potential value for future food research and the food industry, particularly those seeking novel ingredients with abundant nutritional content, appealing sensory characteristics, and health-promoting features. In addition, the presented research also concerns the storage aspect, which is extremely important in the context of food safety, but also its durability during the storage and distribution process. To conclude, the purpose of this new investigation is to evaluate the possibility of using persimmon (Dk) fruits mixed with apples for designing innovative products. Physico-chemical parameters, polyphenol composition, antioxidant activity (CUPRAC, FRAP, ORAC, DPPH^•^, ABTS^•+^ assays), and estimation of inhibition on targeted digestive enzymes (α-amylase, α-glucosidase, and pancreatic lipase) were performed after processing (T0), and after 3 (T3) and 6 (T6) months of storage at 20 ± 2 °C in the dark, to achieve this purpose.

## 2. Materials and Methods

### 2.1. Chemicals and Standards

The standards and chemical substances utilised in this study were the same as in the previous work of Gil et al. [23]. All of them were purchased from Merck (Darmstadt, Germany), Extrasynthese (Genay, France) and TransMIT (Giessen, Germany).

### 2.2. Plant Materials and Persimmon/Apple Smoothies Production

10 kg of fresh *D. kaki* var. Rojo Brillante fruits were collected from the “Melotto” plantation (Villacidro, Sardinia, Italy) at a ripe stage and were not treated with CO_2_. This particular variety of persimmon was chosen due to its physico-chemical purposes and geographical location (Sardinia, Italy) and its excellent nutritional and sensorial qualities. A total of 15 kg of apples cv. Šhampion (*M. domestica*) was acquired at the commercial maturity stage from the LA-SAD SP. Z.O.O. (Borzęcin, Błędów, Poland). Then, 1 kg of strawberry tree fruits (*A. unedo*), 0.3 kg of feijoa flowers (*A. sellowiana*), 0.3 kg of saffron flowers (*C. sativus*), and 5 kg of myrtle berries (*M. communis*) were collected in Sardinia (Italy) following Gil et al. [22,23]. All plant materials were collected in their optimal commercial maturity period (ripe fruit for strawberry-tree and myrtle berries and full flowering stage for feijoa and saffron flowers).

Persimmon fruit puree and apple juice were obtained according to the procedure described by Gil et al. [22], and the smoothie base (Dk/Md) was prepared by mixing apple juice with persimmon fruit puree (75:25, *w*/*w*) according to Gil et al. [23]. Dk/Md was after that mixed in appropriate proportions (*w*/*w*) with other semi-products obtained according to Gil et al. [22]: 95:5 for *M. communis* berry extract (Dk/Md + Mc), *A. unedo* purée (Dk/Md + Au), and *A. sellowiana* flowers (Dk/Md + As), and 99.95:0.05 for *C. sativus* petal juice (Dk/Md + Cs). Then, all products (Table S1) were heated to 100 °C, filled in glass jars (135 mL), pasteurized (10 min at 90 °C), and then cooled to room temperature. The five obtained smoothies were analysed right after processing (T0) and after 3 (T3) and 6 (T6) months of storage at 20 ± 2 °C in the dark to replicate conditions during distribution. All samples were prepared in triplicate and analysed to evaluate mean and standard deviation (SD). For the different analytical assays, smoothie samples were used as it is (physico-chemical parameters) or properly extracted (polyphenolic compounds, antioxidant and digestive enzymes inhibition assays) according to each method reported below.

### 2.3. Sensory Evaluation of Smoothies

The sensory analyses were carried out in a laboratory designed according to ISO 8589:2009 standards [24], and the sensory tests of all obtained products were conducted using a 5° hedonic scale (like very much—5, like slightly—4, neither like nor dislike—3, dislike slightly—2, and dislike very much—1) [22,25]. The fully trained panellists scored the products according to the following criteria: taste, consistency, colour, aroma, and desirability. According to the national laws, no ethical approval was required for this study. The panellists were volunteers, informed about the aim of the study and that they could stop the evaluation at any point.

### 2.4. Determination of the Physico-Chemical Parameters in Obtained Smoothies

All determinations were performed in triplicate. Physico-chemical tests were determined according to Polish norms (PN) [26]: the ash content was determined by the PN-EN 1135:1999 norm and expressed as g per 100 g of fresh weight (fw) [27]; the total soluble solids (TSS) content was evaluated according to PN-EN 12143:2000, and presented as °Brix [28]; titratable acidity (TA) was determined by using PN-EN 12147:2000, and expressed as g of malic acid (MA)/100 g fw [29]; the total content of L-ascorbic acid was determined by the PN-A-04019:1998 norm, and presented as mg in 100 g fw [30]. The smoothies in their original form were used for all of the above analyses.

### 2.5. Determination of Polyphenolic Compounds in Persimmon/Apple Smoothies

Polyphenolic compounds were extracted according to the procedure reported by Gil et al. [23] and Wojdyło et al. [31]. Qualitative (LC/MS QTof) and quantitative (UPLC-PDA) analyses of polyphenolic compounds were carried out following Wojdyło et al. [26] using the UPLC system, Acquity, equipped with a photodiode detector (PDA) and a mass detector G2 QTof Micro mass spectrometer (Waters, Manchester, UK) (Waters Corp., Milford, MA, USA). The polymeric procyanidins were analysed by the phloroglucinol method described previously by Kennedy and Jones [32] using the UPLC system, Acquity, with a fluorescence detector (FL). The final results were presented as mg per 100 g fw.

### 2.6. Determination of Total Phenolic Content, Total Reducing Power, Free Radical Scavenging Activity, and Digestive Enzymes Inhibition Assays

All smoothies for analysis of pro-health properties were prepared in the same way as described in the subchapter above (Section 2.5). The total polyphenolic content (TP) was evaluated by using the Folin-Ciocalteu method [33] with slight modifications and presented as mg of gallic acid equivalent (GAE) per 100 g fw. The CUPRAC assay was performed in compliance with the procedure of Bektaşǒglu et al. [34] with modification, and the final results were presented as mmol of Fe^2+^ in 100 g fw. The FRAP, ORAC, ABTS^•+^, and DPPH^•^ assays were performed according to the procedure of Benzie and Strain [35], Ou et al. [36], Re et al. [37], and Tuberoso et al. [33], respectively. The final results for all of the above methods were expressed as mmol Trolox/100 g fw. The TP, FRAP, ABTS^•+^, and DPPH^•^ assays were measured spectrophotometrically, while the ORAC method was measured spectrofluorometrically by a microplate reader Synergy^TM^ H1 (BioTek, Winooski, VT, USA), all of them in triplicate.

The α-glucosidase and α-amylase inhibitory power of the smoothies were based on the Worthington methods [38], assayed following Nowicka et al. [39], while the inhibition power of pancreatic lipase was performed as described previously by Podsędek et al. [40], with minor modifications. The inhibition power of the above-mentioned enzymes was determined in triplicate by a microplate reader Synergy^TM^ H1 (BioTek, Winooski, VT, USA), and the results were expressed as IC_50_.

### 2.7. Statistical Analysis

All statistical analyses were conducted using Statistica 13.3 (StatSoft, Krakow, Poland). Significant differences (*p* ≤ 0.05) between means were evaluated by a one-way ANOVA (results of sensory evaluation), two-way ANOVA (physico-chemical parameters, polyphenolic compounds, and pro-health properties), and Duncan’s multiple-range test. A correlation analysis was performed, and observed differences were performed by using Spearman coefficients of correlation.

## 3. Results and Discussion

For this study, a particular variety of persimmon, *D. kaki* Thunb. var. Rojo Brillante was chosen. It is an astringent persimmon that has excellent nutritional and sensorial qualities and is well adapted to the Mediterranean Basin countries [41]. As soon as produced (T0), the Dk/Md smoothie and other four smoothies obtained with the addition of *M. communis*, *A. unedo*, *A. sellowiana*, and *C. sativus* extracts were evaluated for a set of quali-quantitative parameters, and the polyphenol profile and biological activities were followed during storage for six months after the production.

### 3.1. Sensory Evaluation of Smoothies

**Figure 1:**
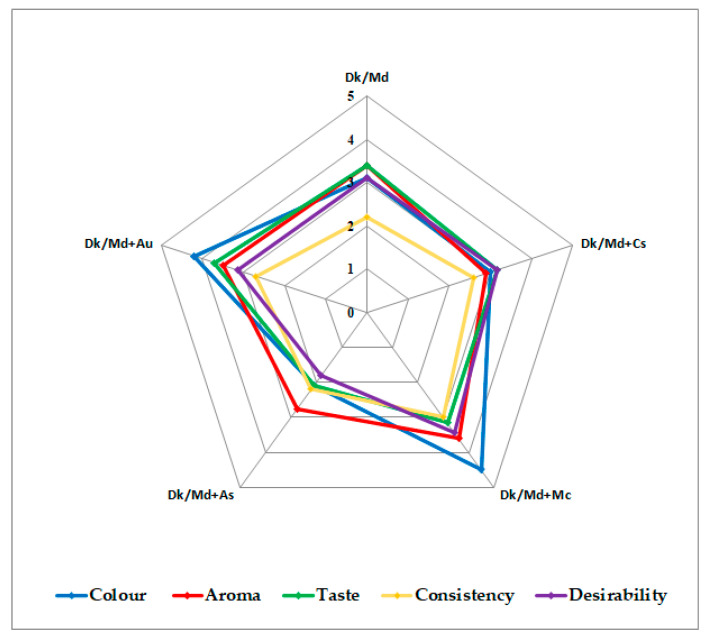
Sensory evaluation of the smoothies (5° hedonic scale).

A daily diet is expected to provide sufficient nutrition to satisfy individuals’ metabolic requirements and give consumers a feeling of well-being and satisfaction through self-indulgent attributes such as taste, aroma, colour, and consistency [42]. The sensory results obtained by the trained panel were grouped according to complex sensory properties: taste, aroma, colour, consistency, and desirability (Table S2). Sensory evaluation was carried out in the smoothies immediately after processing (T0) using a 5° hedonic scale (Figure 1). Based on the results of the study, significant differences (*p* ≤ 0.05) were found between all smoothies for all five evaluated parameters.

The study showed that almost all products were attractive in colour terms (≥3.00). However, the highest colour scores (≥4.20) were obtained in products enriched with strawberry tree fruits (Dk/Md + Au) and myrtle berry extract (Dk/Md + Mc). Only the product with additional feijoa flowers (Dk/Md + As) was not well accepted by the sensory panel regarding the colour (score 2.10). According to consumers, Dk/Md, Dk/Md + Mc, and Dk/Md + Au had the best aroma (≥3.40), while the worst aroma had Dk/Md + As and Dk/Md + Cs (scores of 2.75 and 2.90, respectively). For taste value, the products that came out the best (≥3.40) were Dk/Md and Dk/Md + Au, the smoothie enriched with strawberry tree fruits. The one that tasted the worst (≤2.10) was Dk/Md + As. According to the sensory panel, consistency obtained the lowest scores among all measured parameters. The highest consistency evaluation (score of 3.00) was in products with myrtle berry extract (Dk/Md + Mc). Other products obtained scores between 2.20–2.70. The low scores of consistency were probably due to the already quite dense base (Dk/Md). Furthermore, the additional plant semi-products (Ac, Au, and Cs) made it even more thick. Thus, it would be favourable to prepare a Dk/Md smoothie with a higher percentage of apple juice to obtain a thinner product, which will be better accepted by the end consumer. According to the sensory panel, the best smoothie was Dk/Md + Mc (score of 3.45), followed by Dk/Md + Cs and Dk/Md + Au (score of 3.15). A similar trend of acceptance was observed for apple juices enriched with the same floral semi-products [22]. Products containing feijoa flowers were unacceptable (score of 1.80), confirming what was previously observed in apple juices enriched with this floral semi-product [22]. 

### 3.2. Determination of the Physico-Chemical Parameters

**Figure 2:**
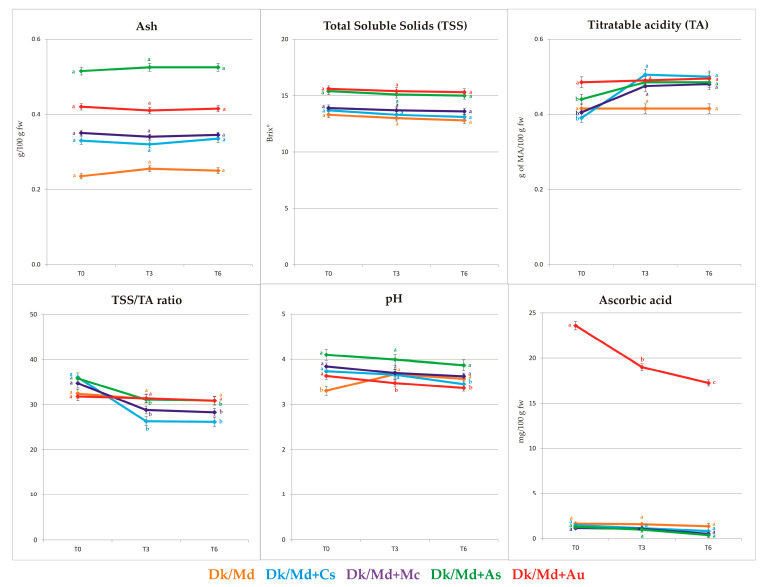
Physico-chemical parameters at T0, T3, and T6. Mean values for each smoothie within a line with different letters (a–c) are significantly different (homogenous groups) at *p* ≤ 0.05).

Ash, total soluble solids (TSS), total acidity (TA), pH, and vitamin C were evaluated in all smoothies immediately after processing and after storage time (three and six months) at 20 ± 2 °C. Statistical differences (*p* ≤ 0.05) were found among all products, and the obtained results are presented in Table S3 and Figure 2. Tables S4–S6 report the Spearman correlation coefficients (*p* ≤ 0.05 and *p* ≤ 0.01) among the physico-chemical parameters and the other investigated parameters.

Differences in ash content between analysed products were observed and reflected in their chemical composition. The highest values of ash were observed in all products, Dk/Md + As and Dk/Md + Au (0.52 and 0.42 g/100 g fw, T0, respectively). Thus, it was observed that all products enriched with feijoa flowers and strawberry tree fruits were the richest in minerals. In contrast, the lowest ash content (0.24 g/100 g fw) was detected in the Dk/Md smoothie. Taking storage into account, no significant (*p* ≥ 0.05) changes were observed among analysed smoothies. Ash showed a significant correlation with TSS (>0.95, T0, Table S4(b)) and showed the same trend during storage (>0.85, Tables S5(b) and S6(b)).

The TSS was also evaluated in this study because it is a characteristic that largely defines the final dry matter content [43]. It was reported [44] that the value of the soluble solid depends on the sugar and organic acids content, as well as on soluble compounds such as tannins, and its value is higher in products richer in these compounds. The highest content of soluble solids was found in Dk/Md + As and Dk/Md + Au (15.40–15.60 °Brix, T0). On the other hand, in other smoothies (Dk/Md + Cs and Dk/Md + Mc), the content of soluble solids was significantly lower (13.70–13.90 °Brix, T0). Nevertheless, each semi-product showed to bring additional value to the pure base (Dk/Md), increasing its content in TSS. During storage, a slight decrease (1–4%) in TSS was observed in analysed final products. According to Kheiralipour et al. [45] and Zatylny et al. [46], the total solid content depends not just on the cultivar but may also be influenced by many other factors such as the degree of fruit dehydration, harvest time, climatic and agricultural conditions and an increase in the insoluble solids’ content of the fruit during maturation.

Significant differences were observed among both pH and TA values in all smoothies. Moreover, some significant differences (*p* ≤ 0.05) were observed in the final products during storage (three and six months). The highest content of titratable acidity was determined in Dk/Md + Au and Dk/Md + As (0.49 and 0.44 g of MA/100 g fw, T0, respectively). In other smoothies, TA was at a comparable level, from 0.39 to 0.44 g of MA/100 g fw, T0. The high content of organic acids in *A. unedo* berries was also confirmed by Oliveira et al. [47]. While *A. sellowiana* flowers have never been investigated before for their organic acid content, their profile and content were similar to that of feijoa fruits [48]. Nevertheless, it was confirmed by Gil et al., 2023 [22] that smoothies enriched with feijoa flowers had a significantly higher amount of total organic acids compared to pure 100% apple juice. Therefore, these two semi-products proved responsible for the acidity of the final smoothies. Low total acidity is important in terms of technological preservation because of possible problems in the pasteurisation process during juice preparation at a pH below or above 4.6 [49].

It is well known that TSS and TA and their ratio are used as quality indicators and parameters indicating the consumer’s preferences [50,51]. Moreover, according to Jaros et al. [51], there are some consumers that, in general, prefer sweeter juices with higher ratios of TSS/TA. It was partially confirmed in this study that the panel of consumers preferred sweeter smoothies but with lower TSS/TA ratios in general.

The pH of all analysed smoothies at T0 varied from 3.31 to 4.10, while during storage time, it decreased slightly: 3.47–3.99 (T3) and 3.37–3.87 (T6) in products enriched with semi-products. In turn, the pH of Dk/Md slightly increased during the storage (3.67 and 3.57, T3 and T6, respectively). These results suggest that enriching smoothie Dk/Md in selected semi-products may lower the pH value and prolong the shelf-life of the prepared smoothies. The addition of the same selected plant materials semi-products to apple juice [22] showed a very similar trend, with the apple juice enriched with feijoa flowers reaching a pH of 4.00.

Analysing the content of ascorbic acid (vitamin C), it was observed that strawberry tree fruits significantly enriched Dk/Md with this vitamin (23.59 mg/100 g fw T0). The high content of ascorbic acid in *A. unedo* fruits was also confirmed in the findings of Morgado et al. [52]. In contrast, the addition of Cs, Mc, and As decreased the amount of ascorbic acid (1.13–1.43 mg/100 g fw, T0) compared to Dk/Md (1.60 mg/100 g fw, T0). At T0, a significant correlation was found between ascorbic acid and total acidity (Spearman correlation 0.8793) (Table S4(b)). During storage, ascorbic acid decreased in all smoothies, and at T6, the loss ranges from 17.8% for Dk/Md to 70.8% for Dk/Md + As (17.26 mg/100 g fw).

### 3.3. Identification and Quantification of Polyphenolic Compounds and Analysis of Polymeric Proanthocyanidins by Phloroglucinol Method

The qualitative identification of polyphenolic compounds by LC-PDA-QTof/MS analysis of extracts from five persimmon-apple fruit smoothies was conducted in negative and positive ion modes. The LC/MS analysis of all products revealed the presence of 74 compounds in total, including 9 anthocyanins, 24 hydroxybenzoic acids, 5 hydroxycinnamic acids, 2 dihydrochalcones, 5 flavan-3-ols, and 5 flavonols (Table S7). It was observed that each additional semi-product enriched Dk/Md with different phenolic compounds, characteristic of each raw material. All hydroxycinnamic acid, dihydrochalcones, and flavan-3-ols were detected in all investigated smoothies.

**Figure 3:**
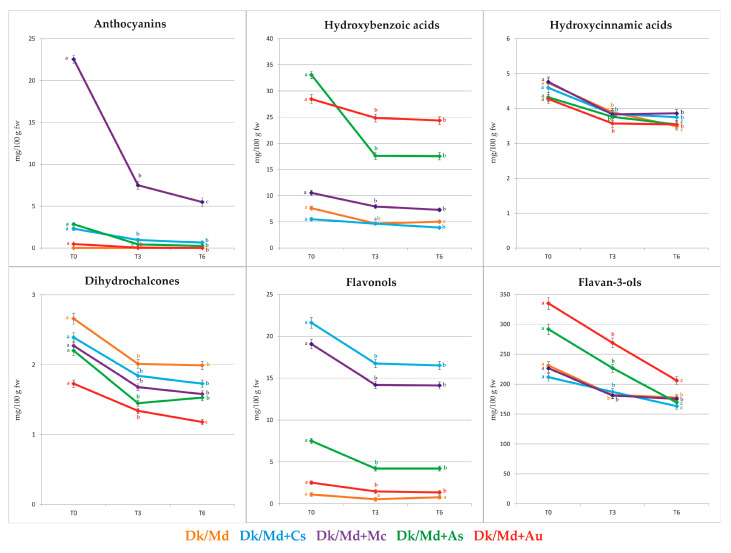
Polyphenolic Compounds at T0, T3, and T6. Mean values for each smoothie within a line with different letters (a–c) are significantly different (homogenous groups) at *p* ≤ 0.05).

The quantification of polyphenols was performed by the UPLC-PDA method (Table S8, Figure 3), and for more sensitive identification of proanthocyanidins (PAC), the quantitative analysis was performed using the UPLC-FL method. Statistically significant differences (*p* ≤ 0.05) were observed between the obtained results of all analysed smoothies immediately after processing and during storage time. The sum of polyphenols evaluated by UPLC-PDA analysis varied in all investigated smoothies, due to a wide concentration range depending on particular plant material. Depending on the type of plant material additive, the total quantity of detected polyphenols in final products (0 months) varied and was the lowest in Dk/Md (247.03 mg/100 g fw) and the highest in Dk/Md + Au (372.34 mg/100 g fw). Generally, based on the analysis of total polyphenols, it was possible to see some trends. It was observed that supplementing the final products with additional components increases the total content of these compounds. 

The Dk/Md smoothie contained many polyphenols, except anthocyanins. Apple and persimmon guaranteed the presence of different hydroxybenzoic acids, such as galloyl glucoside II and III, syringic and salicylic acid (5.15, 2.37, 0.04, and 0.06 mg/100 g fw, respectively), hydroxycinnamic acids, such as neochlorogenic, chlorogenic, caffeic, *p*-coumaric, and *p*-coumaroyloquinic acid (0.25, 3.53, 0.24, 0.08, and 0.63 mg/100 g fw, respectively), dihydrochalcones, such as phloretin-2’-*O*-xyloglucoside and phloretin-2’-*O*-glucoside (1.29 and 1.37 mg/100 g fw, respectively), flavan-3-ols, such as procyanidin B1, B2, and C1, as well as (+)-catechin and (-)-epicatechin (0.90, 1.08, 0.58, 1.19, and 3.76 mg/100 g fw, respectively). Finally, pure base (Dk/Md) was rich in a few flavonols, such as quercetin-3-*O*-galactoside, -glucoside, -arabinoside, -xyloside, and -rhamnoside.

Apart from the polyphenolic profile of pure base, it was possible to identify other compounds deriving from each additional component (strawberry tree fruits, saffron petal juice, purple myrtle berry extract, and feijoa flowers). As previously observed, the LC-PDA-QTof/MS analysis of Dk/Md did not show the presence of anthocyanins. Therefore, the presence of these compounds in analysed smoothies was due to their enrichment with the other plant materials. The total amount of anthocyanins in these smoothies ranged between 2.30 to 22.54 mg/100 g fw. Strawberry tree fruits enriched base with two anthocyanins (cyanidin-3-*O*-galactoside and -arabinoside, 0.41 and 0.07 mg/100 g fw, respectively), while myrtle berry extract enriches it in four anthocyanins unique to this semi-product (delphinidin-pentose, petunidin-3-*O*-glucoside, peonidin-3-*O*-glucoside, and malvidin-3-*O*-glucoside, 0.31, 0.61, 0.03, and 10.96 mg/100 g fw). Furthermore, delphinidin-3,5-*O*-diglucoside was a unique anthocyanin in a smoothie enriched with saffron petal juice (Dk/Md + Cs, 1.76 mg/100 g fw). Additionally, two other anthocyanins (delphinidin-3-*O*-glucoside and cyanidin-3-*O*-glucoside) found in Dk/Md + Mc (8.42 and 2.21 mg/100 g fw, respectively) were also found in Dk/Md + Cs (0.57 mg/100 g fw) and Dk/Md + As (2.84 mg/100 g fw), respectively. Comparison with smoothies obtained with strawberry tree fruit and apple juice enriched with the same selected plant materials semi-products described in this experimentation confirmed those results and the fact that *M. communis* extract can enrich the base in anthocyanins about 20-fold [23].

Strawberry tree fruits additionally enriched the final product with another 20 polyphenols. Except for anthocyanins, among these detected compounds were eight hydroxybenzoic acid derivatives (gallic acid glucoside I and II, galloyl glucoside I, 3-*O*-galloylquinic acid, gallic acid 4-*O*-β-D-glucopyranoside, galloyl shikimic acid, digalloyl shikimic acid, and digalloyl quinic acid, 1.09, 2.19, 2.18, 21.30, 0.06, 0.58, 0.27, and 0.79 mg/100 g fw). Another polyphenol unique to strawberry tree fruits that was found in Dk/Md + Au was quercetin galloylhexose (0.10 mg/100 g fw).

The smoothie enriched with feijoa flowers contained nine hydroxybenzoic derivatives exclusively from this semi-product. Among these were castalagin, casuarin, ellagitannin I and III, nilocitin, casuarinin, ellagic acid, and its two pentosides (arabinoside and xyloside) in the following amounts: 2.29, 0.95, 7.75, 0.95, 1.02, 2.98, 0.62, 0.19, and 3.13 mg/100 g fw, respectively. In addition, three hydroxybenzoic acid derivatives were detected only in smoothies enriched with myrtle berry extract (Dk/Md + Mc). Among these were galloyl-HHDP-glucose I and II, digalloyl-HHDP-glucose I, and ellagitannin II (7.33, 2.36, and 0.84 mg/100 g fw, respectively). In juice with the addition of saffron petal juice, no additional hydroxybenzoic acid derivatives were detected. Therefore, the presence of these compounds in Dk/Md + Cs was due to apple juice and persimmon purée. Furthermore, a few additional hydroxybenzoic acids were found among the analysed smoothies. Galloyl glucoside II and galloyl glucoside III were additionally found in Dk/Md + Cs and Dk/Md + As. The first compound was detected in the feijoa smoothie in double the amount (10.06 mg/100 g fw) compared with the pure base. Syringic acid was detected in this smoothie (Dk/Md + As) as well in an amount almost 10 times greater (0.39 mg/100 g fw) compared to Dk/Md. Both smoothies, with strawberry tree fruits and feijoa flowers, were the richest in hydroxybenzoic acids (28.46 and 33.08 mg/100 g fw, respectively) among all analysed smoothies in this study. 

The average total amount of dihydrochalcones was similar in all analysed smoothies, except for Dk/Md + Au, which showed the lowest amount, even during the storage period. The amount of both detected compounds (phloretin-2’-*O*-xyloglucoside and phloretin-2’-*O*-glucoside, 0.90 and 0.83 mg/100 g fw, respectively) and their total amount was the lowest (1.73 mg/100 g fw) in this smoothie comparing with other analysed smoothies. In particular, smoothies obtained with strawberry tree fruit and apple juice enriched with the same selected plant materials semi-products described in this experimentation reported values of the total amount of dihydrochalcones higher than 3 mg/100 g fw [23]. For flavan-3-ols, some peculiarities were noticed among the smoothies. Dk/Md + Mc was significantly richer than the other smoothies in procyanidin B1 and B2 (10.31 and 4.62 mg/100 g fw, respectively), while (+)-catechin was found in c.a. 2.5 higher amount in Dk/Md + As and Dk/Md + Au (4.39 and 4.02 mg/100 g fw, respectively). Furthermore, regarding polymeric proanthocyanidins, it was observed that only Dk/Md + Au and Dk/Md + As smoothies had higher amounts than Dk/Md (325.63, 281.04, and 223.40 mg/100 g fw, respectively) (Table S8). During storage polymeric proanthocyanidins amount decreases at a different percentage rate (from 23.5% for Dk/Md and Dk/Md + Cs to 36.5% for Dk/Md + Au, T6) and amount in Dk/Md + Au smoothie at T6 was 202.22 mg/100 g fw followed by Dk/Md smoothie with 175.16 mg/100 g fw (Table S8).

Flavonols were a group of polyphenols identified in all tested smoothies. In all smoothies were detected five quercetin-3-*O* derivatives (galactoside, glucoside, arabinoside, xyloside, and rhamnoside), in a following range of 0.29–0.99, 0.07–0.39, 0.05–1.26, 0.11–0.53, and 0.34–1.34 mg/100 g fw, respectively. Unique to the smoothie enriched with saffron petal juice were eight flavonols. The mass spectrometric characterization of compounds provided evidence for the presence of three kaempferol derivatives: -3-*O*-sophoroside-7-*O*-glucoside, -3,7-*O*-diglucoside, and -3-*O*-sophoroside. UPLC-PDA allowed the detection of compounds in the following amounts: 1.81, 0.12, and 11.34 mg/100 g fw. Another five flavonols, particularly for Dk/Md + Cs, were detected as isorhamnetin derivatives: -3,7-*O*-digalactoside, -3,7-*O*-diglucoside, and -3-O-rutinoside, -3-*O*-sophoroside and -3-*O*-glucoside (0.26, 0.95, 0.18, 0.21, and 0.26 mg/100 g fw, respectively).

In addition, myrtle berry extract enriched smoothie with particular for this raw material flavonols. Among them were myricetin galactoside-gallate (1.19 mg/100 g fw), myricetin-3-*O*-arabinoside (1.09 mg/100 g fw), and myricetin (0.37 mg/100 g fw). In turn, feijoa flowers enriched smoothie with kaempherol-3-*O*-galactoside (0.42 mg/100 g fw), quercetin-pentoxide (0.43 mg/100 g fw), kaempferol-hexoside I and II (0.10 and 0.02 mg/100 g fw, respectively), quercetin (0.04 mg/100 g fw), kaempferol (4.04 mg/100 g fw), and apigenin (0.02 mg/100 g fw). Last but not least, strawberry tree fruits enriched smoothies with quercetin galloylhexose (0.10 mg/100 g fw). Furthermore, both Dk/Md + As and Dk/Md + Au were rich in quercetin derivative I (0.06 and 0.12 mg/100 g fw, respectively). In smoothies Dk/Md + Cs and Dk/Md + As was detected quercetin-3,7-*O*-diglucoside (2.09 and 0.05 mg/100 g, respectively), while in smoothies Dk/Md + Mc and Dk/Md + Au were detected myricetin-3-*O*-galactoside (8.69 and 0.26 mg/100 g fw, respectively), -glucoside (0.40 and 0.03 mg/100 g fw, respectively), and -rhamnoside (5.47 and 0.07 mg/100 g fw, respectively).

Following 3 and 6 months of storage at 20 ± 2 °C, a significant (*p* ≤ 0.05) change was observed in the content of phenolic compounds (Table S8). Generally, after storage, the content of total polyphenols decreased in all the smoothies. The total content of polyphenols in smoothies after three months of storage ranged from 193.12 to 300.09 mg/100 g fw, which was a 19% decrement in the overall quantity of polyphenols present in the analysed smoothies. Moreover, after six months, the total polyphenol content decreased by 28% compared to products in T0 and ranged from 189.03 to 236.41 mg/100 g fw. The highest stability during storage, both after three and six months, was observed in the case of polymeric proanthocyanidins (PP). The same trend was noticed in fruit smoothies investigated by Nowicka et al. [25]. The content of PP after three months of storage ranged from 169.20 to 263.48 mg/100 g fw, while after six months of storage, the content of PP ranged from 160.98 to 202.22 mg/100 g fw. According to the findings of Nowicka and Wojdyło [53], the stability of PP depends on food structure (liquid or semi-liquid form). These compounds are more stable in mixed products than in fruit juices. Probably in mixed products (smoothies), the PP is surrounded by the cell wall structure, which inhibits their degradation. Therefore, the degradation of PP in investigated smoothies was slow. During storage, an outstanding decrease of anthocyanins in a smoothie containing 5% purple myrtle berry extract was noticed. After three months, the total amount of these compounds was c.a. three times lower, while after six months, their total amount dropped to four times less compared to fresh smoothies (T0). It is noteworthy that each additional plant matrix had a positive influence on the phenolic composition of original smoothies, enriching Dk/Md with endowed with potential bioactive properties. 

### 3.4. Determination of Total Phenolic Content, Total Reducing Power, and Free Radical Scavenging Activity

**Figure 4:**
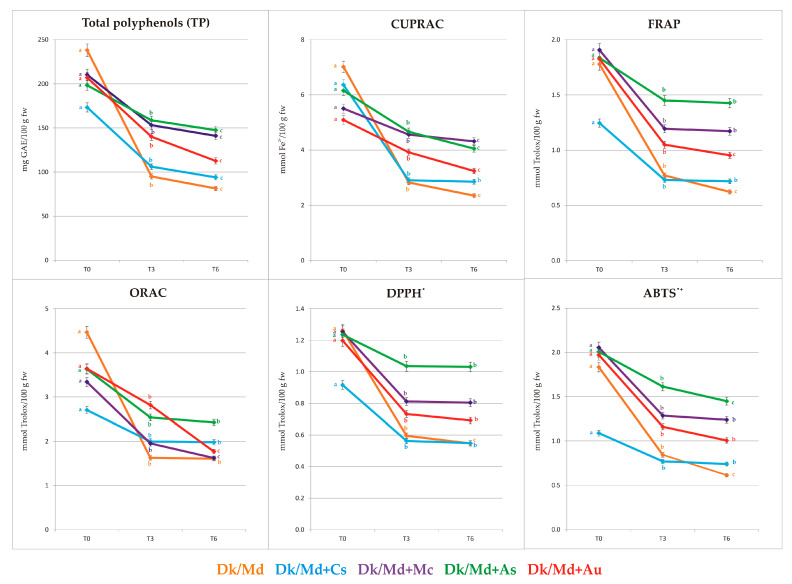
Total phenol content and antioxidant activity at T0, T3, and T6. Mean values for each smoothie within a line with different letters (a–c) are significantly different (homogenous groups) at *p* ≤ 0.05).

Total phenol content (TP) was measured by the Folin-Ciocalteu method, and antioxidant activity of all smoothies was measured by cupric reducing antioxidant activity (CUPRAC), ferric reducing/antioxidant power (FRAP), and free radical-scavenging activity (DPPH^•^ and ABTS^•+^) and oxygen radical absorbance capacity (ORAC) assays. Results of all performed assays (Table S9 and Figure 4) showed approximately the same trends among final products, both before and after storage at room temperature (T3 and T6). A significant (*p* ≤ 0.05) correlation between TP and ORAC assay was found at T0 (0.9369, Table S4(a)), and interestingly, at T3 and T6, an increase in correlations between antioxidant activity and TP (CUPRAC, FRAP, DPPH^•^, and ABTS^•+^) that became highly significant (*p* ≤ 0.01) at T6 with ORAC FRAP, and ABTS^•+^ (Table S6(a)).

Among the analysed final products immediately after processing the highest antioxidant activity (FRAP and ABTS^•+^ method) was determined in Dk/Md + Mc, Dk/Md + As, and Dk/Md + Au in a range from 1.83 to 1.91 mmol Trolox/100 g fw, and 1.97 to 2.06 mmol Trolox/100 g fw. In the case of other performed antioxidant analyses (CUPRAC, ORAC, DPPH^•^) immediately after processing for these three products, the results showed lower values (5.09–6.15 mmol Fe^2+^/100 g fw, 3.34–3.64 mmol Trolox/100 g fw, and 1.20–1.26 mmol Trolox/100 g fw, respectively) compared to pure base. On the other hand, Dk/Md + Cs proved to be less interesting in terms of antioxidant activity than the other smoothies. Although the overall antioxidant activity of analysed smoothies during the storage was decreased, it was observed that all semi-products, except Cs, guaranteed better stabilisation of antioxidant activity. Furthermore, it was observed that at T3 and T6, all products, except Dk/Md + Cs, had a higher antioxidant activity than pure base (Dk/Md). Similar results were observed in the case of total polyphenols content. Immediately after processing, all enriched products showed slightly lower amounts of TP, while after storage, total polyphenol content was always higher in enriched products than in Dk/Md. Greater antioxidant activity in final products was positively associated with a significant increase in total polyphenols content.

At T0, a highly significant correlation (*p* ≤ 0.01) between FRAP and DPPH^•^ and ABTS^•+^ was found (0.9714 and 0.9954, respectively). Moreover, CUPRAC correlated with the total dihydrochalcones amount (0.8970). At T3, most of the antioxidant assays showed significant correlation with each other, and ORAC correlated with most of the phenolic classes detected by UPLC-PDA, but also with DM and ashes (Table S5). Finally, at T6, again, most of the antioxidant assays showed a significant correlation with each other (Table S6(a)).

To sum up, the antioxidant activity depended on the plant materials added to the smoothies and the different stability of the compounds responsible for such activity during ageing. Indeed, according to Ou et al. [36], antioxidant activity depends on the total content of polyphenolic compounds and their types. Similar findings were reported by Gil et al. [22,23], and it was observed that phenolic extracts from smoothies enriched with selected plant materials significantly attenuated the TBH-induced oxidative process in the Caco-2 cells [23]. Moreover, as suggested by Wojdyło et al. [54] and Nowicka et al. [25], antioxidant potential depends on the presence of anthocyanins, flavonols, and polymeric procyanidins. This was also confirmed in the current study. Generally, products rich in anthocyanins (Dk/Md + Mc) and flavan-3-ols, including polymeric procyanidins (Dk/Md + Ac, Dk/Md + Au), were characterised by the highest antioxidant activity. For this reason, adding selected plant material rich in phenolic compounds to smoothies is a strategy for improving their storage stability [18]. Inevitably, TP and antioxidant activity decrease during ageing and slowing down those processes is a crucial challenge that requires optimization of smoothie preparation with proper preservation technologies [16].

### 3.5. Digestive Enzymes Inhibition Assays of Obtained Products before and after Storage

**Figure 5:**
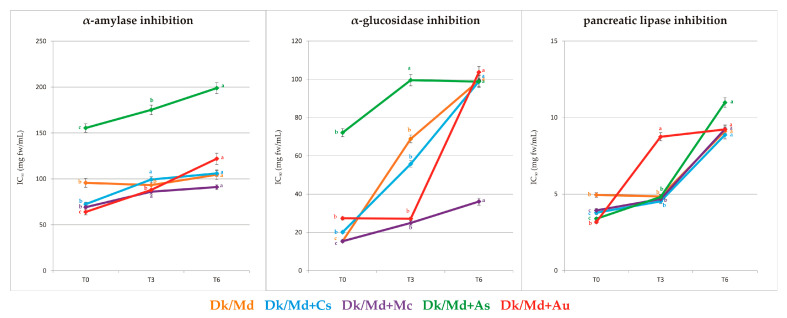
Inhibitory activity against α-amylase, α-glucosidase, and pancreatic lipase at T0, T3, and T6. Mean values for each smoothie within a line with different letters (a–c) are significantly different (homogenous groups) at *p* ≤ 0.05).

Inhibitory activity against α-amylase, α-glucosidase, and pancreatic lipase was measured in all smoothies during a six-month storage period at 20 ± 2 °C, and results were presented as IC_50_ values in Table S10 and Figure 5. In general, significant differences (*p* ≤ 0.05) were found among the analysed final products in inhibitory activities toward these three digestive enzymes immediately after processing and after storage.

The inhibitory activity against α-amylase at T0, presented as IC_50_ values, ranged from 63.98 to 155.46 mg fw/mL, while the values for α-glucosidase were from 15.32 to 72.10 mg of final product/mL. Moreover, the inhibitory activity against pancreatic lipase ranged from 3.17 to 4.94 mg fw/mL. The strongest inhibition of α-amylase was found in smoothies Dk/Md + Au and Dk/Md + Mc in quantities of 63.98 and 69.11 mg fw/mL, respectively. Furthermore, Dk/Md + Cs smoothie was characterised by strong inhibition of α-amylase (72.49 mg of final product/mL). In contrast, the smoothie characterised by the weakest inhibition of α-amylase was a smoothie enriched with feijoa flowers (155.46 mg fw/mL). Generally, those findings agree with Gil et al. [23], except for smoothies enriched with feijoa flowers that were comparable with the others (64.83 mg fw/mL). Time storage negatively affected the inhibitory activity of α-amylase, especially for Dk/Md + Au (9.23 mg fw/mL T6).

Regarding inhibition of α-glucosidase, the strongest IC_50_ values (15.32 mg fw/mL) were observed only for Dk/Md + Mc. Other smoothies (Dk/Md + Cs, Dk/Md + As, and Dk/Md + Au) were characterised by weaker inhibitor activity towards this enzyme (20.08–72.10 mg fw/mL), compared with Dk/Md. The good value of Dk/Md + Mc smoothie confirms the value (15.86 mg fw/mL) reported for the strawberry tree fruit/apple juice smoothies enriched with the *M. communis* semi-product [23].

Inhibition of pancreatic lipase in smoothies ranged from 3.17 to 4.94 mg fw/mL. All enriched products showed to have stronger (c.a. double) inhibition activity than the pure base. The most powerful showed to be a product enriched with myrtle berry extract. Similar values (3.01–3.14 mg fw/mL) were observed in strawberry tree fruit/apple juice smoothies enriched with the same selected plant materials semi-products [23].

Concerning the storage, both α-glucosidase and pancreatic lipase inhibition were negatively affected to a greater extent than α-amylase (Figure 5).

The inhibition of the triglycerides-hydrolysing enzymes (e.g., pancreatic lipase) and carbohydrates-hydrolysing enzymes (e.g., α-amylase and α-glucosidase) can slow the digestion of such nutrients and, consequently, their absorption into the body. This lowers fat intake and postprandial plasma glucose rise, respectively. Long-lasting and slow release of glucose into the bloodstream is particularly crucial in the treatment of hyperglycaemia and type 2 diabetes. Furthermore, inhibition of these digestive enzymes may help to obtain satiety and weight loss in overweight and obese people [39,55].

Among the investigated smoothies was observed strong, as well as moderate inhibition towards tested enzymes. This is favourable for the proper function of the guts and was confirmed by [56]. It is worth noting that the presence of *C. sativus petal juice, A. unedo* fruits, and *M. communis* myrtle berries extract in products has a positive influence on digestive enzyme inhibition. According to Wang et al. [57], inhibition of α-glucosidase is associated with the content of hydroxycinnamic derivatives, such as *p*-coumaric or ferulic acid acids, but in this study, no correlation was found between phenolic acids and inhibition against α-glucosidase. This suggests that other polyphenols (anthocyanins, flavonols, or polymeric procyanidins) might be involved in the inhibitory effect on digestive enzymes. 

To sum up, we can see that some correlation was observed for analysed final products. After enriching the Dk/Md smoothie with other plant additives, polyphenols of Dk/Md interacted with polyphenols contained in other plant materials and, as a consequence, showed high anti-α-glucosidase and anti-α-amylase inhibitory activity, thus creating a highly valuable smoothie with the ability to lower the risk of diabetes.

## 4. Conclusions

Persimmon fruit resulted to be an interesting choice for producing smoothies and enrichment with other plants, especially *A. unedo* and *M. communis*, which enhance the bioactive compounds’ content and biological activities. These mentioned smoothies immediately after processing were characterized by a high content of polyphenolic compounds (372.34 mg/100 g fw, and 285.42 mg/100 g fw, respectively), which directly shaped the high health potential of these products, both in the context of antioxidant and antidiabetic properties. The qualitative analysis of the products obtained clearly indicates that the addition of plant extracts enriches the profile of polyphenolic compounds. Therefore, *M. communis* turned out to be a donor of anthocyanins, especially peonidin-3-*O*-glucoside, and delphinidin-3-*O*-glucoside, the addition of *A. unedo* and *A. sellowiana* fortified the base in hydroxybenzoic acids and polymeric procyanidins, and *C. sativus* resulted in an increase the concentration of flavonols, especially derivatives of isorhamnetin, and quercetin. Thus, the obtained results give grounds to conclude that developing the particular persimmon Rojo Brillante variety may give a chance to revitalize a production sector that in Italy has lost importance in the last decades [58]. Anyway, results show that a six-month period of storage at room temperature causes loss of polyphenols and a decrease of both antioxidant activity and inhibition of targeted digestive enzymes. The amount of polyphenols evaluated by UPLC-PDA analysis decreased in six months from 23.5% for both Dk/Md and enriched *C. sativus* smoothies to 42.5% for enriched *A. sellowiana,* with anthocyanins the most sensitive compounds (71.7–100% loss). Values of CUPRAC, FRAP, ORAC, DPPH^•^, and ABTS^•+^ assays generally strongly decreased during the first three months (up to ca. 60%), and to a lesser extent in the following three months (0.4–27%). In addition, inhibitory activity on α-amylase, α-glucosidase, and pancreatic lipase, especially on the last two enzymes, was negatively affected by time storage. For this reason, better storage strategies to preserve the properties of those smoothies should be developed.

## Data Availability

All related data and methods are presented in this paper. Additional inquiries should be addressed to the corresponding authors.

## Supplementary Materials

The following supporting information can be downloaded at: https://www.mdpi.com/article/10.3390/foods12173248/s1, Table S1. Codes and composition of the smoothies’ samples; Table S2. Sensory evaluation of smoothies (5° hedonic scale); Table S3. Physico-chemical parameters of smoothies before and after storage time (3 and 6 months); Table S4. Spearman correlation coefficients and significance level for T0 smoothies; Table S5. Spearman correlation coefficients and significance level for T3 smoothies; Table S6. Spearman correlation coefficients and significance level for T6 smoothies; Table S7. Qualitative profile of phenolic compounds by LC-PDA/MS QTof method; Table S8. Quantification of phenolic compounds by UPLC-PDA method (mg/100 g fw) in smoothies immediately after processing (T0), after 3 (T3), and 6 months (T6) storage; Table S9. Antioxidant activity of analysed persimmon/apple smoothies before (T0) and after storage (3 and 6 months); Table S10. Digestive enzymes inhibitory activities (IC_50_, mg fw/mL) of analysed persimmon/apple fruit smoothies before (T0) and after storage (3 and 6 months).
