# Supplementary material for: Investigation into Polyphenol Profile and Biological Activities of Enriched Persimmon/Apple Smoothies during Storage
Source: Foods. 2023 Aug 29;12(17):3248. doi: 10.3390/foods12173248 (PMC10486386; doi:10.3390/foods12173248)
Supplement: Supplementary file 1 [file foods-12-03248-s001.zip › foods-2507799-supplementary.pdf]

Supplementary File

Investigation into Polyphenol Profile and Biological Activities of Enriched Persimmon/Apple Smoothies During Storage

Katarzyna Angelika Gil, Paulina Nowicka, Aneta Wojdyło, and Carlo Ignazio Giovanni Tuberoso

Table S1. Codes and composition of the smoothies' samples.

Sample	Content
**Dk/Md**	*Diospyros kaki* fruits puree and *Malus domestica* fruit juice (25:75, w/w)
**Dk/Md+Cs**	Dk/Md + *Crocus sativus* petal's juice (99.95:0.05, w/w)
**Dk/Md+Mc**	Dk/Md + *Myrtus communis* purple berries extract (95:5, w/w)
**Dk/Md+As**	Dk/Md + *Acca sellowiana* flowers (95:5, w/w)
**Dk/Md+Au**	Dk/Md + *Arbutus unedo* fruits puree (95:5, w/w)

Table S2. Sensory evaluation of Smoothies (5° hedonic scale).

Sample	Qualitative parameters				
	Colour	Aroma	Taste	Consistency	Desirability
**Dk/Md**	3.10 ± 0.74 bc	3.4 0± 0.84 a	3.40 ± 0.97 a	2.20 ± 0.63 a	3.10 ± 0.57 a
**Dk/Md+Cs**	3.00 ± 0.82 bc	2.90 ± 0.99 a	3.1 5± 1.06 a	2.60 ± 0.84 a	3.15 ± 0.88 a
**Dk/Md+Mc**	4.50 ± 0.53 a	3.60 ± 0.97 a	3.1 5± 0.94 a	3.00 ± 0.94 a	3.45 ± 0.96 a
**Dk/Md+As**	2.10 ± 0.99 c	2.75 ± 1.18 a	2.1 0± 0.99 a	2.20 ± 1.23 a	1.80 ± 1.03 a
**Dk/Md+Au**	4.20 ± 0.42 ab	3.50 ± 0.85 a	3.70 ± 0.95 a	2.70 ± 0.95 a	3.15 ± 0.94 a

Data are given as mean ± standard deviation (n = 3). Mean values within a column with different letters (a-c) are significantly different (homogenous groups) at $p \leq 0.05$

Table S3. Physico-chemical parameters of smoothies before and after storage time (3 and 6 months).

Sample	0 months (T0)						3 months (T3)						6 months (T6)					
	Ash (g/100 g fw)	TSS (°Brix)	TA (g of MA* /100 g fw)	TSS/TA	pH	Vitamin C (mg/100 g fw)	Ash (g/100 g fw)	TSS (°Brix)	TA (g of MA* /100 g fw)	TSS/TA	pH	Vitamin C (mg/100 g fw)	Ash (g/100 g fw)	TSS (°Brix)	TA (g of MA* /100 g fw)	TSS/TA	pH	Vitamin C (mg/100 g fw)
**Dk/Md**	0.24 ± 0.01 d	13.30 ± 0.03 f	0.42 ± 0.01 cd	32.44 c	3.31 ± 0.03 l	1.60 ± 0.37 d	0.26 ± 0.04 d	13.00 ± 0.05 g	0.42 ± 0.01 cd	31.33 de	3.67 ± 0.00 g	1.54 ± 0.01 d	0.25 ± 0.01 d	12.80 ± 0.01 h	0.42 ± 0.01 cd	30.84 e	3.57 ± 0.00 i	1.32 ± 0.02 def
**Dk/Md+Cs**	0.33 ± 0.00 c	13.70 ± 0.00 e	0.39 ± 0.01 e	36.05 a	3.74 ± 0.01 e	1.43 ± 0.02 de	0.32 ± 0.03 c	13.30 ± 0.02 f	0.51 ± 0.02 a	26.34 g	3.66 ± 0.00 g	1.10 ± 0.24 defg	0.34 ± 0.01 c	13.10 ± 0.03 g	0.50 ± 0.01 ab	26.20 g	3.45 ± 0.00 j	0.80 ± 0.01 gh
**Dk/Md+Mc**	0.35 ± 0.00 c	13.90 ± 0.05 d	0.41 ± 0.01 de	34.75 b	3.84 ± 0.03 d	1.13 ± 0.07 defg	0.34 ± 0.03 c	13.70 ± 0.04 e	0.48 ± 0.02 b	28.84 f	3.70 ± 0.00 f	1.04 ± 0.21 efg	0.35 ± 0.01 c	13.60 ± 0.01 e	0.48 ± 0.01 b	28.33 f	3.62 ± 0.00 h	0.49 ±0.08 hi
**Dk/Md+As**	0.52 ± 0.06 a	15.40 ± 0.00 b	0.44 ± 0.01 c	35.81 a	4.10 ± 0.00 a	1.25 ± 0.24 defg	0.53 ± 0.01 a	15.10 ± 0.12 c	0.49 ± 0.02 ab	31.13 de	3.99 ± 0.01 b	0.96 ± 0.23 fg	0.53 ± 0.02 a	15.00 ± 0.25 c	0.49 ± 0.01 ab	30.93 de	3.87 ± 0.01 c	0.37 ± 0.05 i
**Dk/Md+Au**	0.42 ± 0.02 b	15.60 ± 0.02 a	0.49 ± 0.01 ab	31.84 cd	3.63 ± 0.01 h	23.59 ± 0.60 a	0.41 ± 0.06 b	15.40 ± 0.00 b	0.49 ± 0.00 ab	31.43 de	3.47 ± 0.00 j	19.01 ± 0.05 b	0.42 ± 0.04 b	15.30 ± 0.15 b	0.50 ± 0.01 ab	30.91 de	3.37 ± 0.00 k	17.26 ± 0.34 c

MA* : malic acid; Data are given as mean ± standard deviation (n = 3). Mean values within columns presenting the same characteristics/properties immediately after processing, after 3 months, and after 6 months of storage with different letters (a-l) are significantly different (homogenous groups) at $p \leq 0.05$.

Table S4. Spearman correlation coefficients and significance level for T0 smoothies (see legend at the bottom of the table).

(a)

	Colour	Aroma	Taste	Consistency	Desirability	TP[1] (Folin-Ciocalteus' assay)	CUPRAC	FRAP	ORAC	DPPH•	ABTS•+	α-amylase	α-glucosidase	pancreatic lipase	Total anthocyanins	Total hydroxybenzoic acids
**Aroma**	0.8975															
**Taste**	0.7348	0.7579														
**Consistency**	0.8630	0.5724	0.4344													
**Desirability**	0.8255	0.7632	0.8653	0.6910												
**TP[1]**	0.2094	0.6155	0.2944	-0.2528	0.1565											
**CUPRAC**	-0.6800	-0.4260	-0.2458	-0.7352	-0.2195	0.2065										
**FRAP**	0.3119	0.5238	-0.0621	0.0518	-0.1369	0.6982	-0.3746									
**ORAC**	-0.0799	0.3558	0.1280	-0.5399	-0.1275	0.9396	0.3232	0.6368								
**DPPH•**	0.1686	0.4660	-0.0966	-0.1407	-0.1898	0.8133	-0.1478	0.9714	0.7814							
**ABTS•+**	0.2707	0.4596	-0.1254	0.0410	-0.2077	0.6362	-0.4093	0.9954	0.5941	0.9564						
**α-amylase**	-0.8381	-0.6853	-0.8904	-0.7502	-0.9545	0.0386	0.3994	0.2213	0.2833	0.3212	0.2722					
**α-glucosidase**	-0.6916	-0.7003	-0.8513	-0.5186	-0.9732	-0.2389	-0.0060	0.2080	0.0193	0.2066	0.2896	0.8864				
**pancreatic lipase**	-0.0543	0.2684	0.2354	-0.3045	0.3342	0.6321	0.7490	-0.0178	0.5411	0.1746	-0.1012	-0.0699	-0.5218			
**Total anthocyanins**	0.5698	0.4382	-0.0570	0.7347	0.3702	0.0188	-0.3966	0.3508	-0.2710	0.2425	0.3352	-0.2604	-0.2584	0.0100		
**Total hydroxybenzoic acids**	-0.2302	-0.2484	-0.4194	-0.2343	-0.7006	-0.0540	-0.4658	0.4872	0.1478	0.4058	0.5618	0.5368	0.8093	-0.7219	-0.2513	
**Total hydroxycinnamic acids**	0.2640	0.3745	0.2335	0.2272	0.5879	0.3190	0.4385	-0.0994	0.0763	-0.0090	-0.1772	-0.3397	-0.6864	0.8115	0.5164	-0.8939
**Total dihydrochalcones**	-0.3844	-0.1656	-0.1600	-0.4030	0.0603	0.2677	0.8970	-0.2763	0.2346	-0.0799	-0.3315	0.1891	-0.2573	0.8758	0.0295	-0.7123
**Total flavan-3-ols**	0.5677	0.4759	-0.1074	0.6766	0.2445	0.1285	-0.5013	0.5593	-0.1304	0.4410	0.5530	-0.1493	-0.1126	-0.0870	0.9675	-0.0292
**Polymeric proanthocyanidins**	-0.0145	-0.0069	0.0145	-0.1723	-0.4061	0.0549	-0.5232	0.4024	0.2402	0.3202	0.4550	0.2042	0.5062	-0.6517	-0.4569	0.8890
**Total flavonols**	0.1435	-0.1958	-0.1592	0.5803	0.2831	-0.6962	-0.0922	-0.5318	-0.8639	-0.6223	-0.5225	-0.2892	-0.2235	-0.1351	0.5832	-0.4858

**Total polyphenols (UPLC-PDA)**	0.1108	0.0271	-0.1286	0.0722	-0.4063	-0.0579	-0.7279	0.5168	0.0590	0.3764	0.5812	0.2073	0.5665	-0.8028	-0.1228	0.9348
**Ash**	-0.2988	-0.4697	-0.6411	-0.0723	-0.7374	-0.4139	-0.4816	0.2612	-0.2431	0.1437	0.3529	0.5604	0.8718	-0.8522	-0.0374	0.8963
**TSS[2]**	-0.0692	-0.1865	-0.2652	-0.0320	-0.5414	-0.2149	-0.6381	0.3668	-0.0462	0.2364	0.4440	0.3206	0.6883	-0.8619	-0.2230	0.9617
**TA[3]**	0.2159	0.2677	0.2177	-0.0382	-0.1929	0.2505	-0.5950	0.5438	0.3623	0.4565	0.5754	0.0269	0.2998	-0.5283	-0.3346	0.7875
**TSS[2]/TA[3]**	-0.4766	-0.7191	-0.7355	0.0141	-0.3884	-0.7181	0.1989	-0.4614	-0.6866	-0.4802	-0.4140	0.3860	0.3949	-0.2179	0.2808	-0.0957
**pH**	-0.2827	-0.5420	-0.7874	0.1312	-0.6036	-0.6038	-0.3519	0.0670	-0.5377	-0.0509	0.1491	0.5021	0.7289	-0.6997	0.3330	0.5356
**Vitamin C**	0.4654	0.3959	0.5631	0.2496	0.1924	0.0459	-0.6878	0.2233	0.0815	0.0972	0.2398	-0.4055	-0.0689	-0.5351	-0.3158	0.4933

(b)

	**Total hydroxycinnamic acids**	**Total dihydrochalcones**	**Total flavan-3-ols**	**Polymeric proanthocyanidins**	**Total flavonols**	**Total polyphenols (UPLC-PDA)**	**Ash**	**TSS[2]**	**TA[3]**	**TSS[2]/TA[3]**	**pH**
**Total dihydrochalcones**	0.7771										
**Total flavan-3-ols**	0.3548	-0.1147									
**Polymeric proanthocyanidins**	-0.9119	-0.8137	-0.2465								
**Total flavonols**	0.4015	0.1695	0.3968	-0.6600							
**Total polyphenols (UPLC-PDA)**	-0.8679	-0.9020	0.0920	0.9281	-0.4266						
**Ash**	-0.8114	-0.6525	0.1192	0.6533	-0.0609	0.8109					
**TSS[2]**	-0.9403	-0.8669	-0.0253	0.9155	-0.3613	0.9761	0.8915				
**TA[3]**	-0.7685	-0.8084	-0.1190	0.9609	-0.7157	0.8929	0.4889	0.8212			
**TSS[2]/TA[3]**	0.0914	0.2645	0.1676	-0.4600	0.7624	-0.2635	0.3299	-0.1058	-0.6544		
**pH**	-0.4152	-0.3436	0.3871	0.1564	0.4279	0.4333	0.8455	0.5354	-0.0159	0.7358	
**Vitamin C**	-0.6442	-0.8478	-0.1886	0.8104	-0.4718	0.7208	0.2451	0.6434	0.8793	-0.6827	-0.1958

Legend

TP[1]	Total phenols by Folin-Ciocalteu's assay		significant at $p \leq 0.05$
TSS[2]	Total soluble solids		significant at $p \leq 0.01$
TA[3]	Total acidity		

Table S5. Spearman correlation coefficients and significance level for T3 smoothies (see legend at the bottom of the table).

(a)

	TP[1] (Folin-Ciocalteus' assay)	CUPRAC	FRAP	ORAC	$DPPH^{\bullet}$	$ABTS^{\bullet +}$	α-amylase	α-glucosidase	pancreatic lipase	Total anthocyanins	Total hydroxybenzoic acids	Total hydroxycinnamic acids	Total dihydrochalcones	Total flavan-3-ols	Polymeric proanthocyanidins	Total flavonols	Total polyphenols (UPLC-PDA)
**CUPRAC**	0.9882																
**FRAP**	0.9410	0.9581															
**ORAC**	0.6397	0.5308	0.5652														
**$DPPH^{\bullet}$**	0.8905	0.9115	0.9901	0.5412													
**$ABTS^{\bullet +}$**	0.9303	0.9459	0.9987	0.5843	0.9920												
**α-amylase**	0.4505	0.4655	0.6791	0.3594	0.7736	0.6930											
**α-glucosidase**	-0.0576	-0.0116	0.2548	-0.0443	0.3873	0.2762	0.8538										
**pancreatic lipase**	0.1925	0.0877	0.0482	0.7346	-0.0173	0.0740	-0.2756	-0.4718									
**Total anthocyanins**	0.4329	0.4924	0.2668	-0.2851	0.1735	0.2223	-0.3039	-0.5229	-0.3212								
**Total hydroxybenzoic acids**	0.6143	0.5252	0.5654	0.9611	0.5330	0.5911	0.2820	-0.0828	0.8208	-0.2949							
**Total hydroxycinnamic acids**	-0.4764	-0.3606	-0.3405	-0.9314	-0.2853	-0.3615	-0.0195	0.3235	-0.9270	0.2675	-0.9502						
**Total dihydrochalcones**	-0.5987	-0.4921	-0.3803	-0.8490	-0.2881	-0.3849	0.1389	0.5602	-0.8526	-0.0714	-0.8514	0.9382					
**Total flavan-3-ols**	0.6953	0.7369	0.5327	0.0431	0.4273	0.4984	-0.1732	-0.5259	-0.0440	0.9256	0.0720	-0.0537	-0.3577				
**Polymeric proanthocyanidins**	0.3673	0.2576	0.3280	0.9291	0.3145	0.3610	0.2178	-0.0399	0.8720	-0.5293	0.9543	-0.9493	-0.7844	-0.2023			
**Total flavonols**	0.0277	0.0163	-0.1538	-0.3105	-0.1844	-0.2003	-0.1974	-0.2883	-0.5037	0.5989	-0.5206	0.3863	0.1361	0.3500	-0.5629		
**Total polyphenols (UPLC-PDA)**	0.5520	0.4376	0.4404	0.9753	0.3973	0.4612	0.1610	-0.2121	0.8643	-0.2806	0.9721	-0.9888	-0.9166	0.0500	0.9580	-0.3669	
**Ash**	0.7920	0.7476	0.8613	0.8031	0.8874	0.8735	0.8123	0.4169	0.2204	-0.1640	0.7319	-0.5448	-0.4607	0.1163	0.6175	-0.2134	0.6609
**TSS[2]**	0.7285	0.6456	0.6984	0.9760	0.6771	0.7192	0.4467	0.0333	0.6837	-0.2335	0.9756	-0.8880	-0.8037	0.1258	0.8992	-0.4152	0.9402
**TA[3]**	0.4553	0.3461	0.2701	0.5661	0.2478	0.2506	0.2135	-0.1503	0.1428	0.1167	0.3287	-0.4157	-0.5094	0.1554	0.3120	0.5849	0.4798
**TSS[2]/TA[3]**	0.2425	0.2717	0.4051	0.3643	0.4129	0.4437	0.2542	0.2221	0.4718	-0.3489	0.5863	-0.4108	-0.2335	-0.0537	0.5327	-0.9491	0.4042
**pH**	0.3762	0.4500	0.6120	-0.0517	0.6993	0.6087	0.8834	0.8140	-0.6572	0.0400	-0.1215	0.3934	0.4468	0.0448	-0.2445	0.0485	-0.2582

**Vitamin C**	0.1628	0.0533	0.0032	0.7188	-0.0648	0.0272	-0.3156	-0.5059	0.9979	-0.3123	0.7947	-0.9194	-0.8536	-0.0512	0.8552	-0.4570	0.8529

(b)

	**Ash**	**TSS²**	**TA³**	**TSS²/TA³**	**pH**
**TSS²**	0.8596				
**TA³**	0.5038	0.4383			
**TSS²/TA³**	0.3399	0.5116	-0.5468		
**pH**	0.5511	0.0692	0.0462	0.0625	
**Vitamin C**	0.1852	0.6551	0.1717	0.4163	-0.6935

Legend

TP¹	Total phenols by Folin-Ciocalteu's assay		significant at $p \leq 0.05$
TSS²	Total soluble solids		significant at $p \leq 0.01$
TA³	Total acidity		

Table S6. Spearman correlation coefficients and significance level for T6 smoothies (see legend at the bottom of the table).

(a)

	TP[1] (Folin-Ciocalteus' as-	CUPRAC	FRAP	ORAC	DPPH$^•$	ABTS$^{•+}$	*α*-amylase	*α*-glucosidase	pancreatic lipase	Total anthocyanins	Total hydroxybenzoic	Total hydroxycinnamic	Total dihydro-chalcones	Total flavan-3-ols	Polymeric proanthocya-	Total flavonols	Total polyphe-nols (UPLC-
**CUPRAC**	0.9710																
**FRAP**	0.9787	0.9036															
**ORAC**	0.4654	0.3271	0.6020														
**DPPH$^•$**	0.9313	0.8218	0.9841	0.6610													
**ABTS$^{•+}$**	0.9892	0.9277	0.9964	0.5546	0.9668												
***α*-amylase**	0.5286	0.3305	0.6898	0.9084	0.7823	0.6387											
***α*-glucosidase**	-0.4964	-0.6563	-0.3255	0.4038	-0.2177	-0.3676	0.4194										
**pancreatic lipase**	0.7003	0.5243	0.8286	0.8205	0.9102	0.7813	0.9518	0.1430									
**Total anthocyanins**	0.4947	0.6661	0.3175	-0.3858	0.1984	0.3634	-0.4355	-0.9955	-0.1727								
**Total hydroxybenzoic acids**	0.4220	0.2825	0.4946	0.3263	0.4936	0.5117	0.5389	0.3225	0.4584	-0.3234							
**Total hydroxycinnamic acids**	0.2955	0.5107	0.1187	-0.2252	-0.0273	0.1661	-0.4925	-0.7916	-0.3478	0.8421	-0.4675						
**Total dihydrochalcones**	-0.5187	-0.4867	-0.5031	-0.1972	-0.4171	-0.5524	-0.2687	-0.0147	-0.2182	-0.0195	-0.8746	0.0023					
**Total flavan-3-ols**	0.7084	0.8177	0.5645	-0.2435	0.4647	0.6056	-0.1860	-0.9528	0.0935	0.9421	-0.0452	0.6627	-0.2085				
**Polymeric proanthocyanidins**	-0.1983	-0.2694	-0.1758	-0.3192	-0.1837	-0.1403	-0.0783	0.3660	-0.1960	-0.3748	0.7250	-0.5171	-0.6104	-0.2380			
**Total flavonols**	0.1379	0.3353	0.0040	-0.0109	-0.1166	0.0325	-0.3805	-0.5355	-0.3189	0.6006	-0.5744	0.9269	0.1406	0.3707	-0.6625		
**Total polyphenols (UPLC-PDA)**	0.2521	0.2442	0.2054	-0.2315	0.1210	0.2670	-0.0630	-0.0397	-0.0927	0.0542	0.7966	-0.0821	-0.8914	0.2112	0.8416	-0.3102	
**Ash**	0.7686	0.6402	0.8548	0.8219	0.8583	0.8443	0.8584	0.1585	0.8331	-0.1436	0.7230	-0.1576	-0.6688	0.1072	0.0531	-0.1382	0.3078
**TSS[2]**	0.6038	0.4730	0.6710	0.4833	0.6589	0.6855	0.6449	0.2204	0.5920	-0.2136	0.9692	-0.3285	-0.8925	0.0700	0.5415	-0.4194	0.7165
**TA[3]**	0.4888	0.5266	0.4585	0.4744	0.3476	0.4963	0.2639	-0.0119	0.1765	0.0887	0.4642	0.3800	-0.7539	0.1263	0.0597	0.4307	0.4424
**TSS[2]/TA[3]**	0.1509	-0.0460	0.2684	0.0801	0.3761	0.2430	0.4827	0.3177	0.4986	-0.3897	0.6339	-0.8114	-0.2283	-0.1044	0.5697	-0.9524	0.3482
**pH**	0.6363	0.5239	0.7181	0.6159	0.8018	0.6632	0.7094	-0.1715	0.8632	0.1268	-0.0091	-0.0983	0.2043	0.2993	-0.5676	-0.0889	-0.4551
**Vitamin C**	-0.0975	-0.1423	-0.0922	-0.2109	-0.1356	-0.0458	-0.0573	0.3373	-0.1978	-0.3213	0.7759	-0.3550	-0.7657	-0.2018	0.9599	-0.4738	0.9111

(b)

	**Ash**	**TSS[2]**	**TA[3]**	**TSS[2]/TA[3]**	**pH**
**TSS[2]**	0.8620				
**TA[3]**	0.6649	0.5932			
**TSS[2]/TA[3]**	0.2970	0.5297	-0.3672		
**pH**	0.5053	0.1495	-0.1303	0.2986	
**Vitamin C**	0.1842	0.6328	0.3295	0.3912	-0.6238

Legend

TP[1]	Total phenols by Folin-Ciocalteu's assay		significant at $p \leq 0.05$
TSS[2]	Total soluble solids		significant at $p \leq 0.01$
TA[3]	Total acidity		

Table S7. Qualitative profile of phenolic compounds by LC-PDA/MS QTof method

Code	Compound	R_t (min)	λ_{max} (nm)	MS* [M-H]$^-$(m/z)	MS/MS [M-H]$^-$ (m/z)
Anthocyanins					
A1	Delphinidin-3,5-*O*-diglucoside	3.040	518	627.2785$^+$	465.1909/303.1100
A2	Delphinidin-3-*O*-glucoside	3.856	515	465.1953$^+$	303.1100
A3	Delphinidin-pentose	4.133	520	435.1818$^+$	303.1100
A4	Cyanidin-3-*O*-galactoside	4.135	515	449.1959$^+$	287.1116
A5	Cyanidin-3-*O*-glucoside	4.397	520	449.1959$^+$	287.1116
A6	Cyanidin-3-*O*-arabinoside	4.629	515	419.1797$^+$	287.1116
A7	Petunidin-3-*O*-glucoside	4.648	525	479.2150$^+$	317.1295
A8	Peonidin-3-*O*-glucoside	5.156	525	463.2164$^+$	301.1330
A9	Malvidin-3-*O*-glucoside	5.391	519	493.2339$^+$	331.1495
Hydroxybenzoic acids					
B1	Gallic acid glucoside I	1.156	280	331.1266	271.1605/169.1417
B2	Galloyl glucoside I	1.274	277	331.1334	169.0417
B3	Galloyl HHDP-glucose I	1.325	271	633,1283	481.0828/301.0667
B4	Galloyl glucoside II	1.331	270	331,0639	169.0117
B5	Gallic acid glucoside II	1.366	270	331.1334	271.1990/169.1417
B6	3-*O*-Galloylquinic acid (Theogallin)	1.551	273	343.0742	191.1410
B7	Galloyl HHDP-glucose II	1.591	272	633,1283	481.0828/301.0667
B8	Galloyl glucoside III	1.627	273	331,0639	169.0117
B9	Gallic acid 4-*O*-β-D-glucopyranoside	2.080	320	331.1334	169.0417
B10	Castalagin	2.084	280	933.1019	785.1813/481.0917/301.1057
B11	Galloyl shikimic acid	2.314	272	325.0878	169.0417/125.4180
B12	Casuarin	2.420	374	783.1445	481.0421/301.0667
B13	Digalloylquinic acid	2.755	273	495.1837	343.1255/191.3072
B14	Ellagitannin I	2.829	270	933.1114	781.0445/633.7131/301.1057
B15	Ellagitannin II	3.035	275	783.0645	481.0186/301.1021
B16	Ellagitannin III	3.331	280	783.0759	481.0917/301.0667
B17	Nilocitin	3.653	270	481.0938	301.0667/257.1438
B18	Digalloyl shikimic acid	4.129	278	477.0493	325.0808/169.0417
B19	Syringic acid	4.181	320	197.0458	179.1098/135.0354
B20	Casuarinin	4.391	278	935.0146	765.1799/545.1230
B21	Salicylic acid	5.037	320	136.1212	
B22	Ellagic acid arabinoside	5.703	359	433.0224	301.0631
B23	Ellagic acid xyloside	5.885	361	433.0735	301.0631
B24	Ellagic acid	6.028	366	300.0631	

Hydroxycinnamic acids					
C1	Neochlorogenic acid	3.489	317	353.1287	191.3100/136.0212
C2	Chlorogenic acid	3.723	323	353.0838	191.0534
C3	Caffeic acid	4.036	320	311.0807	179.1098
C4	*p*-Coumaric acid	4.463	323	163.0349	
C5	*p*-Coumaroyloquinic acid	4.904	310	337.0912	163.1014
Dihydrochalcones					
D1	Phloretin-2'-*O*-xyloglucoside	7.433	280	567.1703	273.0757
D2	Phloretin-2'-*O*-glucoside (Phloridzin)	8.186	280	435.1332	273.0733
Flavan-3-ols					
E1	Procyanidin B1	3.256	280	577.1293	289.0708
E2	(+)-Catechin	3.669	280	289.0673	245.0780
E3	(-)-Epicatechin	4.143	280	289.0673	245.0780
E4	Procyanidin B2	4.631	280	577.1293	289.0708
E5	Procyanidin C1	4.956	280	866.1908	577.1188/289.0708
Flavonols					
F1	Kaempferol-3-*O*-sophoroside-7-*O*-glucoside	3.546	346	771.0181	609.0240/2851257
F2	Kaempferol-3,7-*O*-diglucoside	4.636	345	609.0341	447.0543/285.1292
F3	Isorhamnetin-3,7-*O*-digalactoside	4.747	350	639.0883	447.0327/315.0605
F4	Quercetin derivative I	4.766	359	633.0900	463.0633/301.0667
F5	Myricetin galactoside-gallate	4.995	360	631.1107	479.1073/317.1114
F6	Quercetin-3,7-*O*-diglucoside	5.356	352	625.1044	463.0333/301.0924
F7	Myricetin-3-*O*-galactoside	5.460	356	479.0610	317.1114
F8	Myricetin-3-*O*-glucoside	5.534	356	479.0162	317.1114
F9	Isorhametin-3,7-*O*-diglucoside	5.709	343	639.1035	477.1314/315.0948
F10	Quercetin galloylhexose	5.816	360	615.1291	463.0950/301.1092
F11	Kaempferol-3-*O*-sophoroside	5.978	358	609.0240	285.1226
F12	Myricetin-3-*O*-arabinoside	6.027	364	449.0362	317.0678
F13	Isorhamnetin-3-*O*-sophoroside	6.158	352	639.0983	315.0948
F14	Myricerin-3-*O*-rhamnoside	6.239	347	463.0861	317.1114
F15	Quercetin-3-*O*-galactoside	6.345	348	463.0861	301.0994
F16	Quercetin-3-*O*-glucoside	6.494	350	463.1774	301.1447
F17	Kaempferol-3-*O*-galactoside	6.640	347	447.0629	285.1326
F18	Isorhamnetin-3-*O*-rutinoside	6.697	352	623.1223	315.0871
F19	Quercetin-3-*O*-arabinoside	6.765	350	433.0160	301.1421
F20	Quercetin-pentoside	6.957	360	433.0565	301.0959
F21	Quercetin-3-*O*-xyloside	7.063	350	433.0160	301.1421
F22	Quercetin-3-*O*-rhamnoside	7.325	348	447.0148	301.1447
F23	Isorhanetin-3-*O*-glucoside	7.539	352	477.1314	315.0871
F24	Kaempferol-hexoside I	7.568	350	447.1543	285.1326

F25	Myricetin	7.683	367	317.1114	
F26	Kaempferol-hexoside II	7.764	350	447.1543	285.1326
F27	Quercetin	8.774	360	301.1065	
F28	Kaempferol	9.435	360	285.1706	
F29	Apigenin	10.743	360	269.2073	

* $[M+H]^+$ (*m*/*z*) for anthocyanins were obtained in the positive ion mode.

Table S8. Quantification of phenolic compounds by UPLC-PDA method (mg/100 g fw) in smoothies immediately after processing (T0), after 3 (T3) and 6 months (T6) storage.

Code	T0					T3					T6				
	Dk/Md	Dk/Md+Cs	Dk/Md+Mc	Dk/Md+As	Dk/Md+Au	Dk/Md	Dk/Md+Cs	Dk/Md+Mc	Dk/Md+As	Dk/Md+Au	Dk/Md	Dk/Md+Cs	Dk/Md+Mc	Dk/Md+As	Dk/Md+Au
**Anthocyanins**															
A1	nd	1.76±0.16a	nd	nd	nd	nd	0.57±0.06b	nd	nd	nd	nd	0.18±0.01c	nd	nd	nd
A2	nd	0.57±0.11d	8.42±0.22a	nd	nd	nd	0.38±0.02e	2.98±0.11b	nd	nd	nd	0.47±0.02de	2.01±0.10c	nd	nd
A3	nd	nd	0.31±0.03a	nd	nd	nd	nd	nd	nd	nd	nd	nd	nd	nd	nd
A4	nd	nd	nd	nd	0.41±0.01a	nd	nd	nd	nd	0.05±0.00b	nd	nd	nd	nd	nd
A5	nd	nd	2.21±0.12b	2.84±0.10a	nd	nd	nd	0.67±0.04c	0.44±0.03d	nd	nd	nd	0.67±0.05c	0.24±0.02e	nd
A6	nd	nd	nd	nd	0.07±0.00a	nd	nd	nd	nd	nd	nd	nd	nd	nd	nd
A7	nd	nd	0.61±0.01a	nd	nd	nd	nd	0.23±0.02b	nd	nd	nd	nd	0.16±0.01c	nd	nd
A8	nd	nd	0.03±0.00a	nd	nd	nd	nd	0.01±0.00b	nd	nd	nd	nd	nd	nd	nd
A9	nd	nd	10.96±0.32a	nd	nd	nd	nd	3.60±0.12b	nd	nd	nd	nd	2.64±0.11c	nd	nd
**Total**	**nd**	**2.30±0.12e**	**22.54±0.11a**	**2.84±0.10d**	**0.48±0.01h**	**nd**	**0.95±0.05f**	**7.49±0.10b**	**0.44±0.03h**	**0.05±0.00j**	**nd**	**0.65±0.02g**	**5.49±0.11c**	**0.24±0.02i**	**nd**
Hydroxybenzoic acids															
B1	nd	nd	nd	nd	1.09±0.02a	nd	nd	nd	nd	1.05±0.11a	nd	nd	nd	nd	0.90±0.06b
B2	nd	nd	nd	nd	2.19±0.03a	nd	nd	nd	nd	2.10±0.22a	nd	nd	nd	nd	1.80±0.20b
B3	nd	nd	7.33±0.12a	nd	nd	nd	nd	6.41±0.22b	nd	nd	nd	nd	6.05±0.20c	nd	nd
B4	5.15±0.03d	5.06±0.02d	nd	10.06±0.36a	nd	4.14±0.22fg	4.26±0.11ef	nd	7.77±0.32c	nd	4.48±0.20e	3.90±0.24g	nd	8.90±0.34b	nd
B5	nd	nd	nd	nd	2.18±0.03a	nd	nd	nd	nd	2.26±0.32a	nd	nd	nd	nd	2.03±0.06b
B6	nd	nd	nd	nd	21.30±0.56a	nd	nd	nd	nd	18.50±0.41b	nd	nd	nd	nd	18.05±0.21b
B7	nd	nd	2.36±0.11a	nd	nd	nd	nd	0.49±0.02b	nd	nd	nd	nd	0.48±0.02b	nd	nd
B8	2.37±0.19b	0.45±0.03e	nd	2.77±0.12a	nd	0.47±0.02e	0.39±0.02e	nd	1.31±0.06c	nd	0.46±0.03e	0.64±0.06d	nd	1.27±0.13c	nd
B9	nd	nd	nd	nd	0.06±0.00a	nd	nd	nd	nd	0.03±0.00b	nd	nd	nd	nd	0.03±0.00b
B10	nd	nd	nd	2.29±0.22a	nd	nd	nd	nd	1.26±0.03b	nd	nd	nd	nd	1.31±0.15b	nd
B11	nd	nd	nd	nd	0.58±0.08b	nd	nd	nd	nd	0.37±0.02c	nd	nd	nd	nd	0.96±0.02a

B12	nd	nd	nd	0.95±0.05a	nd	nd	nd	nd	0.50±0.03b	nd	nd	nd	nd	0.41±0.02c	nd
B13	nd	nd	nd	nd	0.27±0.03b	nd	nd	nd	nd	0.17±0.02c	nd	nd	nd	nd	0.39±0.02a
B14	nd	nd	nd	7.75±0.42a	nd	nd	nd	nd	1.24±0.02b	nd	nd	nd	nd	1.18±0.01c	nd
B15	nd	nd	0.84±0.09b	nd	nd	nd	nd	1.02±0.21a	nd	nd	nd	nd	0.76±0.11b	nd	nd
B16	nd	nd	nd	0.95±0.15a	nd	nd	nd	nd	0.40±0.02b	nd	nd	nd	nd	0.40±0.02b	nd
B17	nd	nd	nd	1.02±0.10a	nd	nd	nd	nd	0.27±0.01b	nd	nd	nd	nd	0.13±0.00c	nd
B18	nd	nd	nd	nd	0.79±0.12a	nd	nd	nd	nd	0.37±0.03b	nd	nd	nd	nd	0.20±0.02c
B19	0.04±0.00d	nd	nd	0.39±0.03a	nd	0.03±0.00e	nd	nd	0.28±0.03b	nd	0.04±0.00dd	nd	nd	0.22±0.01c	nd
B20	nd	nd	nd	2.98±0.33a	nd	nd	nd	nd	1.68±0.12b	nd	nd	nd	nd	0.09±0.00c	nd
B21	0.06±0.02a	nd	nd	nd	nd	0.04±0.02a	nd	nd	nd	nd	0.05±0.03a	nd	nd	nd	nd
B22	nd	nd	nd	0.62±0.11a	nd	nd	nd	nd	0.34±0.05b	nd	nd	nd	nd	0.35±0.01b	nd
B23	nd	nd	nd	0.19±0.02b	nd	nd	nd	nd	0.15±0.03b	nd	nd	nd	nd	0.23±0.00a	nd
B24	nd	nd	nd	3.13±0.12b	nd	nd	nd	nd	2.42±0.22c	nd	nd	nd	nd	4.05±0.02a	nd
**Total**	**7.62±0.04h**	**5.51±0.06j**	**10.53±0.10f**	**33.08±0.11a**	**28.46±0.13b**	**4.68±0.03l**	**4.65±0.08l**	**7.92±0.06g**	**17.62±0.16e**	**24.85±0.16c**	**5.03±0.06k**	**3.90±0.26m**	**7.29±0.15i**	**17.54±0.22e**	**24.36±0.31d**
Hydroxycinnamic acids															
C1	0.25±0.03cdef	0.19±0.01fg	0.30±0.02bcd	0.31±0.01bc	0.23±0.01efg	0.23±0.01efg	0.22±0.01efg	0.18±0.11g	0.35±0.02ab	0.21±0.02fg	0.28±0.02cde	0.23±0.02efg	0.25±0.02def	0.40±0.02a	0.23±0.01efg
C2	3.53±0.22a	3.48±0.34a	3.44±0.03a	3.09±0.22b	3.13±0.11b	2.87±0.05bc	2.89±0.03bc	2.89±0.12bc	2.64±0.22cd	2.60±0.11cd	2.68±0.10cd	2.80±0.22bc	2.84±0.24bc	2.43±0.11d	2.55±0.23cd
C3	0.24±0.03cde	0.28±0.01abcd	0.31±0.05ab	0.34±0.11a	0.26±0.01bcde	0.25±0.03bcde	0.19±0.03e	0.21±0.01e	0.33±0.01a	0.21±0.02e	0.19±0.01e	0.20±0.02e	0.21±0.03de	0.30±0.01abc	0.21±0.02e
C4	0.08±0.00c	0.08±0.01c	0.15±0.01a	0.05±0.00e	0.15±0.00a	0.05±0.00e	0.07±0.00cd	0.07±0.01cd	nd	0.15±0.01a	0.06±0.00de	0.07±0.00cd	0.10±0.01b	nd	0.15±0.01a
C5	0.63±0.12a	0.57±0.06ab	0.56±0.12abc	0.53±0.02abcd	0.50±0.08bcde	0.49±0.03bcde	0.47±0.06bcde	0.48±0.01bcde	0.44±0.02de	0.40±0.02e	0.28±0.03f	0.45±0.02de	0.46±0.03cde	0.41±0.02e	0.40±0.03e
**Total**	**4.73±0.08a**	**4.59±0.03a**	**4.76±0.06a**	**4.32±0.05b**	**4.27±0.08b**	**3.89±0.06c**	**3.84±0.10c**	**3.83±0.08c**	**3.76±0.05c**	**3.57±0.10d**	**3.49±0.12d**	**3.75±0.10c**	**3.86±0.15c**	**3.54±0.13d**	**3.54±0.20d**
Dihydrochalcones															
D1	1.29±0.06a	1.32±0.08a	1.15±0.04b	1.09±0.12bc	0.90±0.22ef	1.03±0.03bcde	1.05±0.01bcd	0.90±0.01ef	0.97±0.00cde	0.74±0.04g	0.96±0.05cde	0.91±0.05def	0.78±0.03fg	0.76±0.01g	0.65±0.06g
D2	1.37±0.02a	1.07±0.03bcd	1.13±0.10b	1.11±0.02bc	0.83±0.09e	0.98±0.05d	0.79±0.01e	0.78±0.02e	0.78±0.06e	0.60±0.08f	1.03±0.06cd	0.82±0.01e	0.80±0.05e	0.77±0.03e	0.53±0.02f
**Total**	**2.66±0.08a**	**2.39±0.06b**	**2.27±0.10bc**	**2.20±0.04c**	**1.73±0.15fg**	**2.01±0.04d**	**1.84±0.03e**	**1.68±0.02gh**	**1.75±0.03fg**	**1.34±0.10j**	**1.99±0.06d**	**1.73±0.05fg**	**1.58±0.11hi**	**1.53±0.04i**	**1.18±0.08k**
Flavan-3-ols															
E1	0.90±0.10cde	0.82±0.04def	10.31±0.54a	1.20±0.03c	1.12±0.04cd	0.50±0.05fgh	0.55±0.02efgh	8.10±0.44b	0.56±0.08efgh	0.75±0.03efg	0.36±0.03h	0.45±0.02gh	7.91±0.12b	0.85±0.01def	0.77±0.02efg

E2	1.19±0.12f	0.93±0.03g	1.68±0.21e	4.39±0.22a	4.02±0.13b	0.36±0.04i	0.62±0.03h	0.91±0.24g	2.26±0.06c	2.44±0.04c	0.32±0.02i	0.19±0.01i	0.64±0.03h	2.03±0.04d	2.32±0.08c
E3	3.76±0.22a	3.53±0.11ab	3.60±0.42a	3.74±0.10a	3.28±0.12b	1.85±0.12c	1.46±0.03de	1.49±0.03de	1.81±0.24c	1.62±0.11cd	1.41±0.02de	1.24±0.04e	1.39±0.03de	1.43±0.11de	0.34±0.02f
E4	1.08±0.12c	0.96±0.06d	4.62±0.12a	0.83±0.02e	0.56±0.03f	0.06±0.00j	0.14±0.00j	1.18±0.02b	0.24±0.07i	0.36±0.01gh	0.42±0.02g	0.27±0.04ki	1.06±0.04c	0.23±0.01i	0.27±0.02i
E5	0.58±0.02b	0.56±0.03b	0.66±0.05a	0.50±0.04c	0.26±0.00d	0.02±0.00i	0.07±0.00gh	0.12±0.00f	0.27±0.02d	0.16±0.01e	0.07±0.02gh	0.05±0.00hi	0.09±0.01fg	0.25±0.02d	0.06±0.00gh
**Total**	**7.51±0.41i**	**6.80±0.35ij**	**20.87±0.24h**	**10.66±0.09i**	**9.24±0.64i**	**2.79±0.21j**	**2.84±0.08j**	**181.00±1.87d**	**226.56±4.15b**	**268.81±2.34a**	**177.74±2.11de**	**163.18±1.75g**	**175.47±4.54e**	**169.41±3.68f**	**205.98±4.22c**
**PP***	223.40±9.53d	204.72±5.63e	205.39±4.91e	281.04±11.22b	325.63±8.64a	179.22±3.37fg	184.32±2.11f	169.20±5.41ghi	221.42±8.12d	263.48±2.54c	175.16±2.54fgh	160.98±2.11i	164.38±5.41hi	164.62±8.12hi	202.22±6.81e
DP§	7.54	6.95	7.75	7.82	7.00	61.93	70.33	**73.23**	65.41	165.56	68.61	95.32	55.76	69.89	176.40
Flavonols															
F1	nd	1.81±0.22a	nd	nd	nd	nd	1.60±0.03b	nd	nd	nd	nd	1.60±0.05b	nd	nd	nd
F2	nd	0.12±0.01b	nd	nd	nd	nd	0.13±0.00b	nd	nd	nd	nd	0.17±0.00a	nd	nd	nd
F3	nd	0.26±0.26a	nd	nd	nd	nd	0.17±0.03a	nd	nd	nd	nd	0.19±0.02a	nd	nd	nd
F4	nd	nd	nd	0.06±0.00c	0.12±0.01a	nd	nd	nd	0.04±0.00e	0.06±0.00c	nd	nd	nd	0.07±0.00b	0.05±0.00d
F5	nd	nd	1.19±0.02a	nd	nd	nd	nd	0.86±0.05b	nd	nd	nd	nd	0.69±0.04c	nd	nd
F6	nd	2.09±0.12a	nd	0.05±0.00d	nd	nd	1.75±0.06b	nd	0.05±0.00d	nd	nd	1.66±0.11c	nd	0.05±0.00d	nd
F7	nd	nd	8.69±0.34a	nd	0.26±0.04c	nd	nd	6.54±0.12b	nd	0.22±0.01d	nd	nd	6.68±0.15b	nd	0.20±0.01d
F8	nd	nd	0.40±0.01a	nd	0.03±0.00e	nd	nd	0.31±0.01c	nd	0.02±0.00f	nd	nd	0.35±0.01b	nd	0.05±0.00d
F9	nd	0.95±0.11a	nd	nd	nd	nd	0.72±0.02b	nd	nd	nd	nd	0.72±0.03b	nd	nd	nd
F10	nd	nd	nd	nd	0.10±0.00a	nd	nd	nd	nd	0.04±0.00c	nd	nd	nd	nd	0.06±0.00b
F11	nd	11.34±0.24a	nd	nd	nd	nd	9.38±0.22b	nd	nd	nd	nd	9.21±0.22b	nd	nd	nd
F12	nd	nd	1.09±0.03a	nd	nd	nd	nd	0.66±0.02c	nd	nd	nd	nd	0.72±0.03b	nd	nd
F13	nd	0.18±0.00b	nd	nd	nd	nd	0.10±0.00c	nd	nd	nd	nd	0.21±0.02a	nd	nd	nd
F14	nd	nd	5.47±0.24a	nd	0.07±0.00c	nd	nd	4.45±0.20b	nd	0.04±0.00c	nd	nd	4.33±0.15b	nd	0.03±0.00c
F15	0.31±0.02d	0.29±0.03de	0.62±0.14b	0.99±0.12a	0.45±0.02c	0.20±0.01ef	0.17±0.01f	0.49±0.02c	0.65±0.03b	0.26±0.01def	0.23±0.02def	0.21±0.01ef	0.49±0.03c	0.67±0.05b	0.25±0.02def
F16	0.07±0.00g	0.39±0.03a	0.17±0.01d	0.28±0.02b	0.16±0.01de	0.02±0.00h	0.23±0.01c	0.13±0.01f	0.14±0.01ef	0.08±0.01g	0.06±0.00g	0.28±0.01b	0.14±0.01ef	0.16±0.01de	0.08±0.00g
F17	nd	nd	nd	0.42±0.02a	nd	nd	nd	nd	0.24±0.02b	nd	nd	nd	nd	0.26±0.02b	nd
F18	nd	0.21±0.03c	nd	nd	nd	nd	0.79±0.03b	nd	nd	nd	nd	0.95±0.04a	nd	nd	nd
F19	0.14±0.01cd	1.26±0.12a	0.05±0.00ef	0.19±0.01c	0.07±0.00def	0.03±0.01f	0.84±0.04b	0.04±0.00ef	0.10±0.00def	0.04±0.00ef	0.05±0.00ef	0.15±0.12cd	0.05±0.00ef	0.12±0.00cde	0.04±0.00ef
F20	nd	nd	nd	0.43±0.03a	nd	nd	nd	nd	0.20±0.02b	nd	nd	nd	nd	0.21±0.02b	nd

F21	0.21±0.02d	0.11±0.01fg	0.17±0.02e	0.53±0.06a	0.47±0.04b	0.11±0.01fg	0.05±0.00h	0.08±0.00gh	0.29±0.01c	0.26±0.02c	0.14±0.02ef	0.11±0.00fg	0.06±0.00h	0.28±0.02c	0.21±0.01d
F22	0.38±0.03efg	1.34±0.20a	0.84±0.05bc	0.34±0.03fgh	0.79±0.04c	0.17±0.02j	0.64±0.00d	0.49±0.02e	0.22±0.01ij	0.45±0.03ef	0.30±0.03ghi	0.91±0.07b	0.43±0.02ef	0.24±0.01hij	0.38±0.02fg
F23	nd	0.26±0.01a	nd	nd	nd	nd	0.16±0.02b	nd	nd	nd	nd	0.14±0.01b	nd	nd	nd
F24	nd	nd	nd	0.10±0.00a	nd	nd	nd	nd	0.05±0.00c	nd	nd	nd	nd	0.07±0.00b	nd
F25	nd	nd	0.37±0.02a	nd	nd	nd	nd	0.14±0.01c	nd	nd	nd	nd	0.19±0.01b	nd	nd
F26	nd	nd	nd	0.02±0.02a	nd	nd	nd	nd	0.01±0.01a	nd	nd	nd	nd	0.02±0.02a	nd
F27	nd	nd	nd	0.04±0.00a	nd	nd	nd	nd	0.02±0.00b	nd	nd	nd	nd	0.02±0.00b	nd
F28	nd	nd	nd	4.04±0.24a	nd	nd	nd	nd	2.20±0.12b	nd	nd	nd	nd	2.04±0.14c	nd
F29	nd	nd	nd	0.02±0.00a	nd	nd	nd	nd	nd	nd	nd	nd	nd	nd	nd
**Total**	**1.11±0.03k**	**21.61±0.08a**	**19.06±0.10b**	**7.51±0.08f**	**2.52±0.03h**	**0.53±0.02m**	**16.73±0.08c**	**14.19±0.06e**	**4.21±0.10g**	**1.47±0.04i**	**0.78±0.02l**	**16.51±0.12d**	**14.13±0.09e**	**4.21±0.03g**	**1.35±0.06j**
**Total phenolic compounds (including polymeric proanthocyanidins)**															
	**247.03±5.32f**	**247.92±4.12f**	**285.42±5.26d**	**341.65±2.12b**	**372.34±3.22a**	**193.12±2.33jk**	**215.17±2.11h**	**216.11±3.15h**	**254.34±2.51e**	**300.09±4.12c**	**189.03±1.02k**	**189.72±2.13k**	**207.81±0.87i**	**196.46±0.45j**	**236.41±1.22g**

Data are given as mean ± standard deviation (n = 3). Mean values within a line with different letters (a-n) are significantly different (homogenous groups) at $p \leq 0.05$. * PP: polymeric proanthocyanidins, quantitative data of were obtained using the phloroglucinol method; § DP: Degree of polymerisation.

Table S9. Antioxidant activity of analysed persimmon/apple smoothies before (T0) and after storage (3 and 6 months).

Sample	0 months (T0)						3 months (T3)						6 months (T6)					
	TP†	CUPRAC†‡	FRAP‡	ORAC‡	DPPH‡	ABTS•+‡	TP†	CUPRAC‡	FRAP‡	ORAC‡	DPPH‡	ABTS•+‡	TP†	CUPRAC‡	FRAP‡	ORAC‡	DPPH‡	ABTS•+‡
**Dk/Md**	238.04 ± 10.16a	7.01 ± 0.07a	1.78 ± 0.16b	4.47 ± 0.28a	1.26 ± 0.02a	1.83 ± 0.04d	94.98 ± 2.92gh	2.82 ± 0.02i	0.77 ± 0.02h	1.63 ± 0.11g	0.59 ± 0.02g	0.84 ± 0.03l	81.34 ± 5.52i	2.35 ± 0.02j	0.62 ± 0.01i	1.61 ± 0.20g	0.55 ± 0.01g	0.61 ± 0.00n
**Dk/Md+Cs**	173.23 ± 8.73c	6.36 ± 0.05b	1.25 ± 0.02d	2.70 ± 0.01de	0.92 ± 0.02d	1.09 ± 0.02j	106.08 ± 6.70fg	2.91 ± 0.13i	0.73 ± 0.00h	1.99 ± 0.05f	0.56 ± 0.05g	0.77 ± 0.00m	93.96 ± 4.23h	2.85 ± 0.20i	0.72 ± 0.02h	1.98 ± 0.10f	0.55 ± 0.01g	0.74 ± 0.00m
**Dk/Md+Mc**	210.20 ± 10.41b	5.50 ± 0.11c	1.91 ± 0.04a	3.34 ± 0.21c	1.25 ± 0.07ab	2.06 ± 0.02a	153.13 ± 9.11d	4.56 ± 0.14e	1.19 ± 0.02d[illegible]	1.95 ± 0.18f	0.81 ± 0.01e	1.28 ± 0.01g	140.95 ± 3.90e	4.31 ± 0.19f	1.17 ± 0.03e	1.62 ± 0.15g	0.80 ± 0.02e	1.24 ± 0.02h
**Dk/Md+As**	198.20 ± 5.37b	6.15 ± 0.04b	1.83 ± 0.01b	3.63 ± 0.22b	1.24 ± 0.03ab	2.01 ± 0.02b	158.58 ± 7.88d	4.66 ± 0.26e	1.45 ± 0.01c	2.54 ± 0.16de	1.04 ± 0.02c	1.61 ± 0.03e	147.37 ± 6.38de	4.05 ± 0.14g	1.43 ± 0.03c	2.42 ± 0.05e	1.03 ± 0.03c	1.45 ± 0.03f
**Dk/Md+Au**	207.01 ± 5.62b	5.09 ± 0.09d	1.83 ± 0.01b	3.64 ± 0.27b	1.20 ± 0.04b	1.97 ± 0.03c	139.96 ± 5.13e	3.92 ± 0.18g	1.05 ± 0.01f	2.82 ± 0.16d	0.73 ± 0.02f	1.16 ± 0.01i	112.58 ± 5.79f	3.24 ± 0.12h	0.95 ± 0.01g	1.77 ± 0.04fg	0.69 ± 0.01f	1.01 ± 0.01k

†mg GAE/100 g fw; ‡ mmol Trolox/100 g fw; †‡ mmol Trolox/100 g fw. Data are given as mean ± standard deviation (n = 3). Mean values within columns presenting the same properties immediately after processing, after 3 months, and after 6 months of storage with different letters (a-n) are significantly different (homogenous groups) at $p \leq 0.05$.

Table S10. Digestive enzymes inhibitory activities (IC_{50}, mg fw/mL) of analysed persimmon/apple fruit smoothies before and after storage(T0) and after storage (3 and 6 months).

Sample	0 months (T0)			3 months (T3)			6 months (T6)		
	†α-amylase	†α-glucosidase	†pancreatic lipase	†α-amylase	†α-glucosidase	†pancreatic lipase	†α-amylase	†α-glucosidase	†pancreatic lipase
**Dk/Md**	95.68 ± 0.03h	15.37 ± 0.03a	4.94 ± 0.11g	93.21 ± 0.11g	68.82 ± 0.12g	4.85 ± 0.01g	104.51 ± 0.15j	99.29 ± 0.01j	9.11 ± 0.11j
**Dk/Md+Cs**	72.49 ± 0.01c	20.08 ± 0.01b	3.78 ± 0.04c	99.34 ± 0.09i	55.75 ± 0.06f	4.52 ± 0.11e	105.89 ± 0.09k	98.81 ± 0.22i	8.87 ± 0.02i
**Dk/Md+Mc**	69.11 ± 0.04b	15.32 ± 0.00a	3.93 ± 0.03d	86.02 ± 0.08d	24.88 ± 0.01c	4.67 ± 0.08f	91.05 ± 0.14f	36.07 ± 0.11e	9.24 ± 0.05j
**Dk/Md+As**	155.46 ± 0.22m	72.10 ± 0.23h	3.38 ± 0.11b	175.19 ± 0.15n	99.51 ± 0.00j	4.81 ± 0.11g	198.85 ± 0.21o	98.80 ± 0.12i	10.97 ± 0.03k
**Dk/Md+Au**	63.98 ± 0.01a	27.32 ± 0.05d	3.17 ± 0.08a	87.92 ± 0.11e	27.10 ± 0.42d	8.74 ± 0.09h	121.97 ± 0.54l	103.71 ± 0.31k	9.23 ± 0.05j

†mg fw/mL. Data are given as mean ± standard deviation (n = 3). Mean values within columns presenting the same characteristics/properties immediately after processing, after 3 months of storage, and after 6 months of storage with different letters (a-o) are significantly different (homogenous groups) at $p \leq 0.05$.
